# Infrared plasmons propagate through a hyperbolic nodal metal

**DOI:** 10.1126/sciadv.add6169

**Published:** 2022-10-26

**Authors:** Yinming Shao, Aaron J. Sternbach, Brian S. Y. Kim, Andrey A. Rikhter, Xinyi Xu, Umberto De Giovannini, Ran Jing, Sang Hoon Chae, Zhiyuan Sun, Seng Huat Lee, Yanglin Zhu, Zhiqiang Mao, James C. Hone, Raquel Queiroz, Andrew J. Millis, P. James Schuck, Angel Rubio, Michael M. Fogler, Dmitri N. Basov

**Affiliations:** ^1^ Department of Physics, Columbia University, New York, NY 10027, USA.; ^2^ Department of Mechanical Engineering, Columbia University, New York, NY 10027, USA.; ^3^ Department of Physics, University of California, San Diego, La Jolla, CA 92093, USA.; ^4^ Max Planck Institute for the Structure and Dynamics of Matter, Center for Free Electron Laser Science, Hamburg 22761, Germany.; ^5^ Università degli Studi di Palermo, Dipartimento di Fisica e Chimica Emilio Segrè, via Archirafi 36, I-90123 Palermo, Italy.; ^6^ Department of Physics, Pennsylvania State University, University Park, PA 16802, USA.; ^7^ 2D Crystal Consortium, Materials Research Institute, Pennsylvania State University, University Park, PA 16802, USA.; ^8^ Center for Computational Quantum Physics (CCQ), Flatiron Institute, New York, NY 10010, USA.

## Abstract

Metals are canonical plasmonic media at infrared and optical wavelengths, allowing one to guide and manipulate light at the nanoscale. A special form of optical waveguiding is afforded by highly anisotropic crystals revealing the opposite signs of the dielectric functions along orthogonal directions. These media are classified as hyperbolic and include crystalline insulators, semiconductors, and artificial metamaterials. Layered anisotropic metals are also anticipated to support hyperbolic waveguiding. However, this behavior remains elusive, primarily because interband losses arrest the propagation of infrared modes. Here, we report on the observation of propagating hyperbolic waves in a prototypical layered nodal-line semimetal ZrSiSe. The observed waveguiding originates from polaritonic hybridization between near-infrared light and nodal-line plasmons. Unique nodal electronic structures simultaneously suppress interband loss and boost the plasmonic response, ultimately enabling the propagation of infrared modes through the bulk of the crystal.

## Teaser

Nodal-line metals allow hyperbolic infrared waveguiding through the bulk with band structure–engineered loss reduction.

## INTRODUCTION

**Fig. 1.**
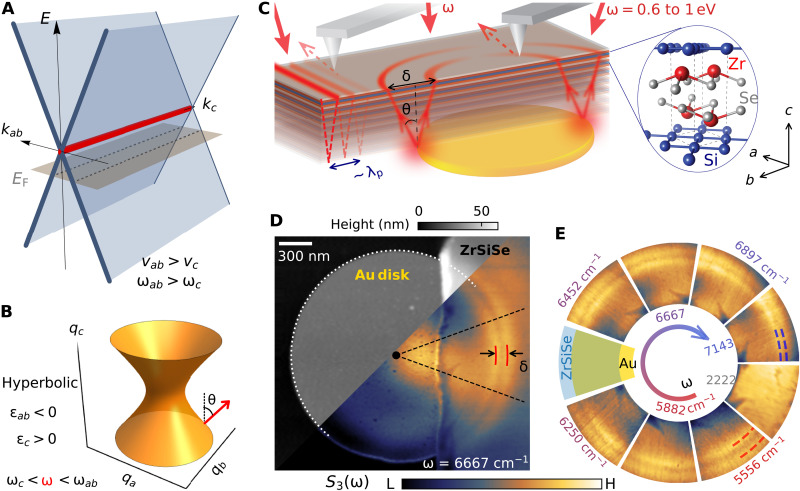
Infrared waveguide modes in nodal-line metal ZrSiSe. (**A**) Schematic band structure *E* versus *k*_ab_, *k*_c_ showing the Dirac nodal line (red). The gray plane indicates the Fermi level. (**B**) Schematic isofrequency surface inside the hyperbolic regime (ε*_ab_* · ε*_c_* < 0) for a nodal-line metal. Red arrow indicates the direction of the group velocity of the hyperbolic ray. (**C**) Schematic of the nanoimaging setup. The near-infrared laser illuminates the sample and hyperbolic plasmon polaritons (HPPs; red lines) are launched by an atomic force microscope (AFM) tip at the edge or by an underlying gold antenna. The AFM-based nano-optics registers the evanescent fields associated with the waveguide modes in the bulk in the form of linear fringes or characteristic rings. The layered crystal structure of ZrSiSe is shown in the inset. (**D**) Topography (gray scale) and near-field scattering amplitude *S*_3_ (color scale) of a 26-nm ZrSiSe crystal partially covering a gold disk. White dotted line indicates the boundary of the Au disk. Red solid lines mark the split center peaks of hyperbolic polariton modes along the circumference. (**E**) Images of *S*_3_ obtained within the sector region indicated by black dashed lines in (D) and assembled for laser frequencies from ω = 7143 to 5556 cm^−1^ within the hyperbolic region. The image taken outside of the hyperbolic range at 2222 cm^−1^ is devoid of the double-ring structure.

Nodal-line semimetals reveal Dirac-like linear dispersion of electronic bands with nodes extending along lines/loops in the Brillouin zone (Fig. 1A) (*1*, *2*). These systems present an appealing platform to investigate quantum effects originating from the interplay of topology, reduced dimensionality, and electronic correlations encoded in unconventional optical responses (*3*, *4*). Here, we focus on the nodal metal ZrSiSe, which hosts nearly two-dimensional electronic structure and high-mobility Dirac fermions (*2*). We show that the nodal band structure and the attendant van Hove singularities (VHSs) suppress the interband transitions (*5*–*7*) and boost plasmonic response, thus enabling propagation of infrared waveguide modes in metallic samples. We use scanning near-field optical microscopy to visualize the nanoscale infrared signatures of waveguide modes and evaluate their energy-momentum (ω, *q*) dispersion.

Common materials bounce light at frequencies where the real part of the dielectric function (ε = ε_1_ + *i*ε_2_) becomes negative. Perhaps counterintuitively, anisotropic media, including layered crystals, do support propagating modes in their interior provided the in-plane and out-of-plane dielectric functions are of opposite sign ($\varepsilon_{1}^{ab}\cdot \varepsilon_{1}^{c}<0$). Because the relevant isofrequency surface (Fig. 1B) is the hyperboloid, these media are referred to as hyperbolic (*8*–*10*). In the hyperbolic regime, the interaction of light with collective modes of crystals yields hyperbolic polaritons imbuing crystals with exotic optical properties, including negative reflection at interfaces (*11*) and ray-like waveguiding in the bulk (*12*, *13*). Hyperbolic waveguiding has mostly been explored in polar insulators inside their narrow phonon bands, including hBN (*14*, *15*), MoO_3_ (*16*, *17*), V_2_O_5_ (*18*), calcite (*19*), Ga_2_O_3_ (*20*), and semiconducting WSe_2_ (*21*). Expanding the spectral bandwidth of hyperbolic polaritons and extending hyperbolicity in the near-infrared frequency range are highly desirable but difficult. Here, we achieved this challenging task by hybridizing light with plasmonic modes of a nodal metal, ZrSiSe.

Hyperbolic waveguiding is anticipated in a wide variety of anisotropic conductors (*8*, *22*–*26*). As the screened plasma frequency ω_p_ marks the zero crossing of ε_1_, a vast frequency range of hyperbolicity appears between $\omega_{p}^{c}<\omega <\omega_{p}^{ab}$, where $\varepsilon_{1}^{ab}<0$ and $\varepsilon_{1}^{c}>0$ (*27*). While anisotropic metals, in principle, offer broadband hyperbolicity, the inherently strong electronic loss (*28*) prevents waveguiding. It is customary to quantify the electronic loss through the complex optical conductivity (σ = σ_1_ + *i*σ_2_). The precondition for propagating polaritons σ_2_/σ_1_ ≫ 1 is rarely fulfilled although the hyperbolic plasmons are reported in various electronic systems. Commonly, interband transitions lead to a large σ_1_ in the near-infrared range and therefore severely limit the plasmon propagation (*28*). Here, we demonstrate a practical route toward a metal with reduced electronic losses by harnessing the nodal band structure and the attendant VHSs. Our data show that the nodal metal ZrSiSe attains a local minimum of σ_1_ near the VHS energy. The sharp reduction in σ_1_ that we observe is accompanied by increases in σ_2_ that collectively lead to the inequality σ_2_/σ_1_ ≫ 1 over a broad frequency range. The enhanced plasmonic properties along with concurrently reduced interband losses allow for direct experimental observation of propagating hyperbolic plasmon polaritons (HPPs) in the near infrared.

## RESULTS

### Antenna-launching experiment

To visualize infrared waveguide modes in ZrSiSe, we performed two types of nanoimaging experiments (Fig. 1C). The first one involved placing thin crystals of ZrSiSe on patterned gold antennas, which served as launchers of hyperbolic rays into the interior of the sample (*12*, *13*, *21*). The second approach used the sample edge to reflect the HPPs and revealed characteristic higher-order hyperbolic modes (*12*, *15*). The two complementary experiments produced consistent results.

We first focus on experiments involving an Au disk launcher underneath the crystal. As illustrated in Fig. 1C, the HPPs propagate as conical rays and emerge on the top surface of the sample as “hot rings” with enhanced nano-optical contrast surrounding the edge of the Au antennas. The propagation angle θ (with respect to surface normal) is controlled by the anisotropic permittivities of the sample (*12*, *13*, *29*)

$$\text{tan}(\theta )=\sqrt{-\varepsilon_{1}^{ab}/\varepsilon_{1}^{c}}=\frac{\delta /2}{d}$$ (1)

where δ is the separation of the rings on the top surface and *d* is the sample thickness. We obtained colocated topography and nano-optical amplitude [*S*_3_(ω); see Materials and Methods] images of a thin ZrSiSe crystal partially covering the gold disk (Fig. 1D). At ω = 6667 cm^−1^, a clear double-ring pattern (Fig. 1D) emerges along the Au antenna boundary.

**Fig. 2.**
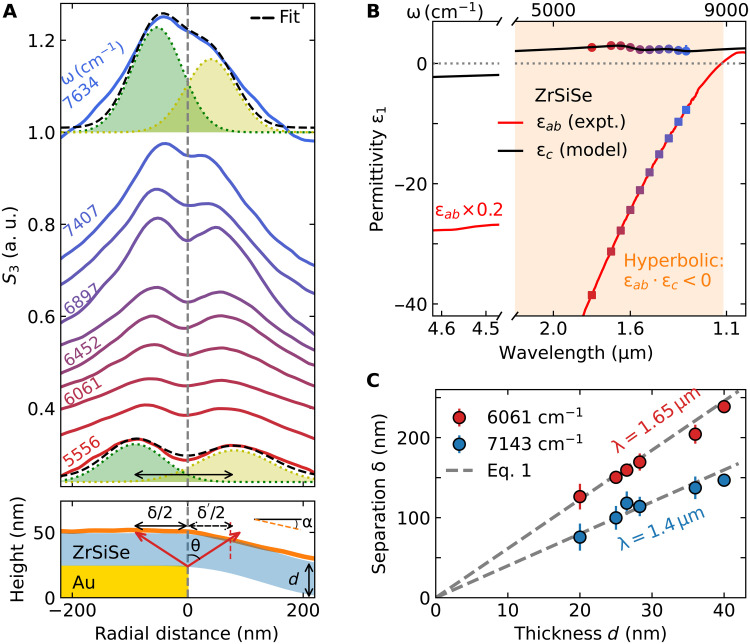
Hyperbolic electrodynamics of ZrSiSe. (**A**) Line profiles of the near-field scattering amplitude *S*_3_ at several incident frequencies. Black dashed lines are fits using Gaussian functions for the ω = 7634 and 5556 cm^−1^ line profiles. Green and yellow shaded areas indicate the individual Gaussian functions representing the hyperbolic ray profiles. Bottom shows the topography line profile (orange) near the edge of the Au disk (gold) in Fig. 1D. The downward slope (tanα) of the sample (blue) leads to a geometrical correction to the measured ring separation δ. (**B**) In-plane dielectric function (ε*_ab_*, red line) obtained from far-field optical measurements (*5*). The ε*_ab_* values at selected frequencies (squares) together with δ(ω) in (A) are used to extract ε*_c_* (circles) using Eq. 1. The black line is a Drude-Lorentz fit of the experimental out-of-plane dielectric function data. (**C**) Hyperbolic ray separation δ(ω) as a function of flake thickness *d* at ω = 6061 cm^−1^ (red) and ω = 7143 cm^−1^ (blue). The separations scale linearly with increasing flake thickness, as prescribed by Eq. 1 (gray dashed line).

This double-ring pattern is confined to the vicinity of the antenna edges and is distinct from the intensity variation in the interior of our structures prompted by the internal resonances of the Au antenna at much longer length scales. In Fig. 1D, the ring separation (δ≈150 nm) is an order of magnitude smaller than the free-space light wavelength (λ = 1.5 μm, ω ≈ 6667 cm^−1^). The double-ring pattern also varies with incident light frequency, as shown in Fig. 1E, where we assemble the *S*_3_(ω) data at selected frequencies. The blue and red dashed lines mark the positions of the hot rings at ω = 7143 and 5556 cm^−1^, with systematic evolution of the ring separation for frequencies in between. In contrast, the double-ring feature is completely absent in the sector for ω = 2222 cm^−1^ outside of the hyperbolic range quantified in Fig. 2; instead, this sector shows a homogeneous near-field response (see also fig. S6).

We now inquire into quantitative details of propagating HPPs in ZrSiSe. We average the radial line profiles within the sectors depicted in Fig. 1E and plot these in Fig. 2A. The experimental ring separation δ(ω) is obtained by fitting the line profile with two Gaussian functions and a linear background, shown for ω = 7634 and 5556 cm^−1^ in Fig. 2A (see text S3 for complete analysis). With the *ab*-plane permittivity known from (*5*), the experimental ring separation δ, together with the sample thickness *d*, allows for the extraction of the *c*-axis permittivity of ZrSiSe from Eq. 1. These latter data are displayed in Fig. 2B along with the experimental *ab*-plane permittivity (squares). The hyperbolic regime in ZrSiSe extends between ≈2837 and 9091 cm^−1^ (see texts S1 and S3). Last, we observe yet another hallmark of the hyperbolic rays, which is the scaling of the interpeak separation δ with increasing sample thickness (Fig. 2C), δ = 2*d*$\sqrt{-\varepsilon_{1}^{ab}/\varepsilon_{1}^{c}}$. Broadband hyperbolic electrodynamics in the layered nodal metal ZrSiSe is therefore firmly established.

### Higher-order hyperbolic polariton modes

**Fig. 3.**
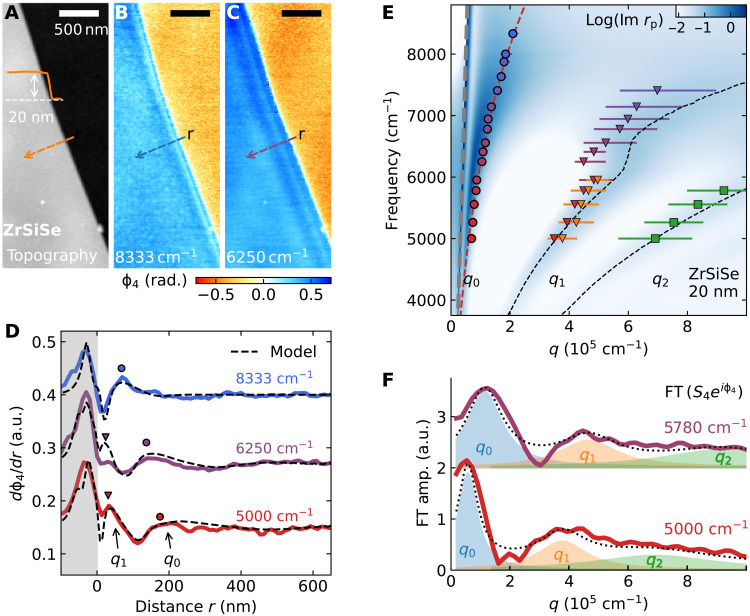
Hyperbolic plasmon polaritons in ZrSiSe. Topography (**A**) and near-field phase (ϕ_4_) image of a 20-nm-thin crystal of ZrSiSe at (**B**) ω = 8333 cm^−1^ and (**C**) ω = 6250 cm^−1^. (**D**) Phase derivative line profiles (*d*ϕ_4_/*dr*) at multiple laser frequencies near edges of ZrSiSe. For ω = 6250 and 5000 cm^−1^, the derivative profiles reveal features at multiple spatial periodicities (*q*_0_ and *q*_1_). Black dashed lines are the simulation of the derivative profile with two periodicities, corresponding to the principal (*q*_0_) and higher-order (*q*_1_) HPPs. a.u., arbitrary units. (**E**) Frequency-momentum dispersion of HPP plotted in the form of Im(*r*_p_). Circles, the principal modes; triangles, higher-order polaritons. Data points are superimposed over the calculated Im(*r*_p_) described in the text. The gray dashed line represents the free-space light cone. Black dashed lines indicate numerical solutions for the divergence of Im(*r*_p_) for the higher-order HPP branches. Red dashed line is a guide for the dispersion of the principal branch. The kink in the experimental dispersion near ω ≈ 6200 cm^−1^ probably originates from the impact of surface states (fig. S28). (**F**) Fourier transform (FT) of the complex near-field signal *S*_4_*e*^*i*ϕ_4_^ along the same path in (B) to (D) at ω = 5780 and 5000 cm^−1^. Multiple peaks in the Fourier transform amplitude correspond to the principal (*q*_0_) and higher-order (*q*_1_, *q*_2_) modes and are fitted by Lorentzian functions (color-shaded area).

The natural edges of thin hyperbolic materials can also launch and reflect polaritons emanating from the metallic tip (Fig. 1C) (*30*). To explore HPPs near the edges, we focused on the phase contrast, which provides highest level of image fidelity (*31*–*33*). The phase-contrast data reveal weak higher-order HPP modes: yet another electrodynamics signature of hyperbolicity (*12*, *15*). In Fig. 3 (A to C), we present the topography and near-field phase-contrast images obtained for a 20-nm-thin ZrSiSe crystal on an Si/SiO_2_ substrate for two representative laser frequencies. At ω = 8333 cm^−1^ (Fig. 3B), the phase contrast displays a prominent fringe near the edge, which shifts further into the interior of the sample as the laser frequency decreases in Fig. 3C. The first peak-dip separation systematically increases in thicker samples (fig. S15). To quantify the HPP wavelength, we used a previously developed electromagnetic solver (*34*) to simulate the phase contrast with the complex polariton momentum *q*_p_ = (1 + *i*γ)2π/λ_p_ as input. Here, λ_p_ is the polariton wavelength, and γ accounts for the damping of the polariton wave.

Multiple fringes of different periodicities appear at lower frequencies and are particularly apparent at ω = 6250 cm^−1^ (Fig. 3C). To better resolve these shorter wavelength oscillations, we inspected the derivative of the phase line profiles, *d*ϕ_4_/*dr*. In Fig. 3D, we show the experimental and simulated phase derivative traces for ω= 8333, 6250, and 5000 cm^−1^ (see fig. S13 for additional data). The profile obtained at ω = 8333 cm^−1^ can be adequately reproduced with a single damped polariton of wavelength λ_p0_ ≈ 300 nm. However, for ω = 6250 and 5000 cm^−1^, an additional mode with a much shorter wavelength λ_p1_ is needed to fully account for the data. Both weaker (*q*_1_) and stronger (*q*_0_) peaks in the derivative line profiles are well described by the simulation (black dashed line) involving two polariton modes with different polaritons of wavelengths λ_p0_ and λ_p1_. The polariton momentum can then be extracted from the wavelength as Re$\;q_{p}=\frac{2\pi }{\lambda p}$, enabling a direct comparison with the theoretical dispersion. As we show below, the two modes correspond to the principal and higher-order HPPs in ZrSiSe and are in accord with the experimental dielectric tensors in Fig. 2B.

The extracted HPP momenta are organized in the dispersion (ω, *q*) plot in Fig. 3E. It is customary to identify HPPs via the divergences of the reflection coefficient *r*_p_(ω, *q*) (*14*). A colormap of Im(*r*_p_) provides an instructive way to visualize both the dispersion and the damping of the HPP modes. The colormap is calculated for a 20-nm-thick crystal of ZrSiSe residing on a SiO_2_/Si substrate using experimental dielectric functions (Fig. 2B). As expected, multiple dispersive branches develop in the hyperbolic frequency range, corresponding to the principal and higher-order modes. The existence of higher-order modes can also be documented by directly Fourier transforming the experimental real-space line profile (*12*, *15*). As shown in Fig. 3F, the Fourier transform amplitudes of the complex signal *S*_4_*e*^*i*ϕ_4_^ for ω= 5780 and 5000 cm^−1^ (see fig. S14 for additional data) indeed display up to three distinct modes that can be parameterized by Lorentzian functions. The obtained momenta (*q*_0_, *q*_1_) are consistent with the values from line profile modeling (Fig. 3, D and E); Fourier transforms are also suggestive of an additional weaker higher-order mode *q*_2_. The calculated hyperbolic dispersions agree with the experimental momenta (colored circles and triangles), unequivocally corroborating the notion of HPPs in ZrSiSe. The deviation of the higher-order branch (*q*_1_) and the data points (triangles) is within the experimental error bars. Nevertheless, this slight discrepancy hints at the presence of surface states in ZrSiSe (*35*–*37*) with potentially different dielectric responses (see text S4) from the bulk values (Fig. 2B) used in our calculation. Furthermore, around 6200 cm^−1^, a kink in the experimental dispersion of the *q*_1_ mode shows more pronounced deviation from the prediction based on bulk dielectric constants. This kink structure can also be attributed to the impact of increased metallicity and additional interband transition from the surface state bands (see fig. S28).

## DISCUSSION

**Fig. 4.**
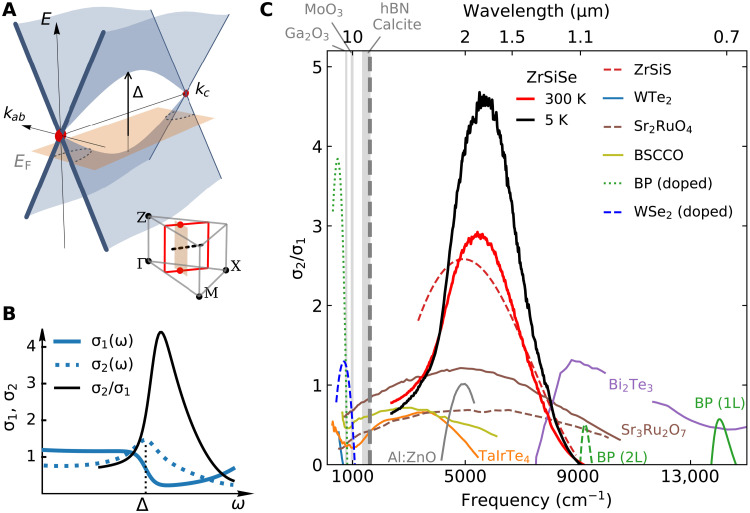
Band structure origin of enhanced plasmonic hyperbolicity in a nodal metal. (**A**) Schematic band structure *E* versus *k_ab_*, *k_c_* inside the nodal square (red line in inset). Vertical arrow indicates the van Hove energy Δ. (**B**) Model for the interband optical conductivity (σ = σ_1_ + *i*σ_2_) of a nodal line near the van Hove energy (see Materials and Methods). The ratio σ_2_/σ_1_ exhibits a maximum near Δ. The optical conductivity is related to the dielectric function via σ = *i*ω(1 − ε)/4π. (**C**) Survey of experimental σ_2_/σ_1_ ratio for representative plasmonic hyperbolic materials [WTe_2_ (*22*, *23*), TaIrTe_4_ (*24*), ZrSiS (*25*), black phosphorus (BP) (*26*), doped WSe_2_ (*21*), Bi_2_Sr_2_CaCu_2_O_8_ (*42*, *43*), Sr_2_RuO_4_ (*44*), Sr_3_Ru_2_O_7_ (*45*), and Bi_2_Te_3_ (*46*)], metamaterial Al:ZnO (*47*), and excitonic hyperbolic materials [few-layer BP (*48*)]. (See figs. S20 to S27 for extracted optical conductivities).

The propagating HPPs observed in ZrSiSe would not have been possible without tamed interband losses: a unique feat of the nodal band structure uncovered by our experiments. In ZrSiSe, the nodal lines form “squares” in momentum space with lines of VHSs inside the nodal squares (Fig. 4A, inset) (*5*, *7*). The resulting saddle-point structure (Fig. 4A) leads to a suppression of interband transitions above the van Hove energy (Δ). The corresponding dissipative part of the conductivity σ_1_(ω) shows a “cliff” above Δ (Fig. 4B), accompanied by a peak in σ_2_(ω) at Δ prescribed by Kramers-Kronig relations. We emphasize that the enhancement in σ_2_(ω) is an important approach toward high-quality factor plasmons (*28*, *34*, *38*). The unique combination of reduced dissipation (σ_1_) and enhanced plasmonic response (σ_2_), quantified by the ratio $\frac{\sigma_{2}}{\sigma_{1}}\approx 3\;\text{to}\;5$ for ZrSiSe (Fig. 4C), is superior to that of all other candidate plasmonic and excitonic hyperbolic materials reported so far. The plasmonic qualities in ZrSiSe are anticipated to be further enhanced at cryogenic temperatures (Fig. 4C, black curve).

The suppression of interband transitions near the van Hove energy offers a novel strategy for the “band structure engineering” approach to mitigating loss and boosting plasmonic response (*39*, *40*). While the existence of “loss-less metal” remains elusive (*28*), we propose that VHSs in topological systems (*41*) reveal as-yet untapped plasmonic design rule afforded by nodal-line semimetals.

## MATERIALS AND METHODS

### Single crystal growth and device fabrication

The ZrSiSe single crystals were synthesized using a chemical vapor transport method as described previously (*2*, *5*). For Au antenna–patterned devices, Au/Cr (25 nm/1 nm) disks were e-beam–deposited on SiO_2_/Si substrates following standard e-beam lithography processes using a lift-off resist. ZrSiSe flakes were then directly exfoliated on Au/Cr disks in a glove box filled with inert gas [O_2_ < 1 part per million (ppm), H_2_O < 0.1 ppm]. Before exfoliation, the substrates were annealed in glove box at 250°C for 1 hour to remove any residual moisture on the surface.

### Near-infrared nano-optical measurements

We used a scattering-type scanning near-field optical microscope (Neaspec) based on an atomic force microscope (AFM) operating in tapping mode. The tapping frequency of the AFM tip is around 70 kHz, and near-field data are collected at higher harmonic (*n* = 3 or 4) of the tapping frequency to suppress the far-field background. For the gold antenna launcher experiment, the difference frequency generation outputs of a pulsed laser source (Pharos, Light Conversion) were used. We used a continuous-wave tunable laser from M Squared to obtain phase contrast images near the edges of thin crystals. Tunable outputs between 1140 and 2200 nm are generated by frequency mixing of a high-power 532-nm diode laser (Equinox) and a Ti:sapphire laser tunable between 700 and 1000 nm (SolsTiS).

### Geometrical correction considering finite antenna thickness

Because of the finite thickness of the underlying Au antenna, the sample exhibits a downward slope outside the antenna boundary (Fig. 2A, bottom). This small slope (tanα) leads to a finite asymmetry ($\frac{\delta }{2}>\frac{\delta \prime }{2}$) in the propagation distance of the two rays (Fig. 2A), which we corrected in the extraction of δ(ω) as following. The two distances δ and δ^′^ are related by $\frac{\delta }{\delta^{\prime }}=1+\text{tan}\alpha \cdot \text{tan}\theta$, where $\text{tan}\theta =\frac{\delta }{2d}$ and *d* is the sample thickness. The measured double-ring distance is denoted as $\Delta =\frac{\delta }{2}+\frac{\delta \prime }{2}$. Substituting δ^′^ in terms of Δ and δ, we obtained $\delta =\Delta -\frac{2d}{\text{tan}\alpha }+\sqrt{\Delta^{2}+\frac{4d^{2}}{{\text{tan}}^{2}\alpha }}$. At the limit of α → 0 (no downward slope) or *d* → ∞ (infinitely thick sample), $\sqrt{\Delta^{2}+\frac{4d^{2}}{{\text{tan}}^{2}\alpha }}\approx \frac{2d}{\text{tan}\alpha }$ and therefore, δ → Δ as expected.

### Model for interband optical conductivity of ZrSiSe near VHS

The minimum in the interband optical conductivity of ZrSiSe (see fig. S1) can be modeled by a step function near the VHSs and a Lorentzian function accounting for transitions at higher energy. Specifically, the step function is expressed as $\sigma_{\text{step}}(\omega )=\frac{\text{tanh}[(-\omega +\Delta )/\Gamma \;]+1}{2}+\sigma_{s}$, where Δ is the van Hove energy, Γ is the step width, and σ_s_ is a constant background. The higher-energy optical transition is described by σ_high_(ω) = − *i*ω[ε_∞_ − 1 + *f*^2^/(ω_0_^2^ − ω^2^ − *i*γω)], where ε_∞_ is the high-frequency dielectric constant; *f*^2^, ω_0_, and γ are the oscillator strength, center frequency, and scattering rate of the Lorentzian peak, respectively. The real part of the interband optical conductivity of ZrSiSe can then be expressed as σ_1_ = σ_step_(ω) + Re [σ_high_(ω)], and the imaginary part σ_2_ are obtained numerically through Kramers-Kronig relations. For modeled σ_1_ and σ_2_ shown in Fig. 4B, the following parameters are used: Δ = 1.25, Γ = 0.06, σ_s_ = 0.1, ω_0_ = 2, *f*^2^ = 0.49, γ = 0.5, and ε_∞_ = 2.5.

## Supplementary Materials

### This PDF file includes:

Supplementary Text Figs. S1 to S28 Table S1 References
