# Supplementary material for: Infrared plasmons propagate through a hyperbolic nodal metal
Source: Sci Adv. 2022 Oct 26;8(43):eadd6169. doi: 10.1126/sciadv.add6169 (PMC9604610; doi:10.1126/sciadv.add6169)
Supplement: Supplementary file 1 — Supplementary Text Figs. S1 to S28 Table S1 References [file sciadv.add6169_sm.pdf]

Science Advances
AAAS

Supplementary Materials for

Infrared plasmons propagate through a hyperbolic nodal metal

Yinming Shao *et al.*

Corresponding author: Yinming Shao, ys2956@columbia.edu; Dmitri N. Basov, db3056@columbia.edu

Sci. Adv. **8**, eadd6169 (2022)
DOI: 10.1126/sciadv.add6169

This PDF file includes:

Supplementary Text
Figs. S1 to S28
Table S1
References

Supplementary Text

Section S1: c-axis dielectric function and optical conductivity of ZrSiS and ZrSiSe

The *ab*-plane complex optical conductivity ($\sigma(\omega) = \sigma_1 + i\sigma_2$) of bulk ZrSiSe is obtained using broadband reflectance spectra combined with spectroscopic ellipsometry (*5*). To study the hyperbolicity the *c*-axis dielectric response is also important. While an optically flat *ac*-surface is not attainable in ZrSiSe, measurements on the large and flat *ac*-surface of a closely related ZrSiS compound indeed reveal a much lower plasma frequency along the *c*-axis (Fig. S1, black dashed line), consistent with recent report (*25*). In order to obtain the *c*-axis dielectric function of ZrSiSe, we utilized the gold antenna launchers and performed near-field imaging experiments as discussed in the main text. Given the knowledge of *ab*-plane dielectric function, the antenna launching experiment allow us to extract the *c*-axis dielectric function based on the double-ring spacing (see Section S3). The extracted *c*-axis screened plasma frequency of ZrSiSe is around 3000 cm^{-1} (Fig. S5), similar to the screened plasma frequency of ZrSiS (Fig. S1).

As mentioned in the main text, the unique nodal-square structure of ZrSiSe offers an effective approach to reducing the electronic loss associated with interband optical transitions. We remark that although van Hove singularities (VHS) appear in many electronic systems (e.g., Weyl semimetals), the impact on the optical conductivity of 3D systems rarely leads to a minimum, due to the large joint density of states (JDOS). For a pair of Weyl nodes, the JDOS scales with frequency as ω^2 and correspondingly, $\sigma_1(\omega) \propto \frac{\text{JDOS}}{\omega} = \omega$. As a result, the van Hove singularity only changes the slope of the linear scaling of σ_1 in a Weyl semimetal (*49*, *50*), in contrast to the minimum observed in ZrSiSe (*5*–*7*). In both ZrSiS and ZrSiSe, *ab-initio* calculations of the band structure have clearly identified the saddle point structures at finite k_z in both ZrSiS (*6*) and ZrSiSe (*5*) to be around 0.4 eV. The similarities of the VHS energy scales in ZrSiS and ZrSiSe can be verified directly with bulk optical conductivity spectra, which sums the contribution of all k_z planes. By comparing experimental optical conductivity with *ab-initio* calculations for ZrSiS (*6*, *7*) and ZrSiSe (*5*), the step minimum of $\sigma_1(\omega)$ (and therefore the enhancement of σ_2/σ_1) in both compounds are found to be originated from the VHS.

Section S2: Transport measurements and carrier density of ZrSiSe

Magnetoresistivity and Hall resistivity of ZrSiSe were performed using a standard four-probe technique in a physical properties measurement system (PPMS, Quantum Design), as shown in Fig. S2. Given the coexistence of both electron and hole-like carriers in ZrSiSe, we adopt a two-band model to estimate the carrier densities and mobilities by simultaneously fitting the measured magnetoresistivity and Hall resistivity data. If the contributions of both electron and hole bands to conductivity are assumed to be additive, the longitudinal resistivity (ρ_{xx}) and transverse resistivity (ρ_{xy}) can be described by:

$$\rho_{xx} = \frac{(n_e\mu_e + n_h\mu_h) + (n_e\mu_e\mu_h^2 + n_h\mu_h\mu_e^2)B^2}{(n_e\mu_e + n_h\mu_h)^2 + \mu_e^2\mu_h^2(n_h - n_e)^2B^2} \cdot \frac{1}{e} \qquad (S1)$$

$$\rho_{xy} = \frac{(n_h\mu_h^2 - n_e\mu_e^2) + \mu_e^2\mu_h^2(n_h - n_e)B^2}{(n_e\mu_e + n_h\mu_h)^2 + \mu_e^2\mu_h^2(n_h - n_e)^2B^2} \cdot \frac{B}{e} \qquad (S2)$$

where n_e (n_h) and μ_e (μ_h) are the density and mobility of these electron and hole bands shown in equation (S1) and (S2), respectively. $B = \mu_0 H$ and e are the magnetic field strength and elementary charge. In Fig. S2, we present the two-band model fit to the ρ_{xx} and ρ_{xy} at T = 100 K at a low-field range since no satisfactory fit can be obtained for the low temperature data. We also note the ρ_{xx} and ρ_{xy} data in the high-field range cannot be fitted with the two-band model, probably due to the quantum effects and high order effect as described in (*2*). The best fits yield the carrier densities of $n_e \sim 4.1 \times 10^{20}$ cm^{-3} and $n_h \sim 1.2 \times 10^{20}$ cm^{-3} and the mobilities of $\mu_e \sim$ 1030 cm^2/Vs and $\mu_h \sim$ 4522 cm^2/Vs, which is consistent with previous reports (*2*).

Section S3: Antenna launching near-field imaging data and fitting

In Fig. S3 we show the full frequency dependence of the antenna launching experimental data in the hyperbolic regime. Since the diameter of the Au disk antenna (2 µm) is comparable to the laser wavelength (1.3 − 1.8 µm), the near-field signal exhibits diffraction patterns inside the Au antenna, as shown in the main text. On the other hand, the ZrSiSe region covering the Au antenna shows an enhancement in near-field amplitude and a gradual increase in the "double-ring" separation. This separation reaches a maximum at $\omega = 5556$ cm^{-1} and the length scale (≈190 nm) is an order of magnitude smaller than the laser wavelength ($\lambda = 1.8$ µm).
To quantify the double-ring separations, we fitted the line profiles of S_3 with two Gaussian functions and a linear background, as shown in Fig. S4. Together with the slope correction discussed in the Materials and Methods section, we extracted the frequency-dependent peak separation $\delta(\omega)$, as shown in Fig. S5. The out-of-plane (*c*-axis) dielectric constant of ZrSiSe is then obtained using the experimental *ab*-plane dielectric constant and $\delta(\omega)$, according to $\sqrt{\varepsilon_c} = \sqrt{-\varepsilon_{ab}}\left(\frac{2d}{\delta}\right)$, where d is the thickness of the ZrSiSe crystal.

The extracted *c*-axis dielectric functions of ZrSiSe are modeled with Drude-Lorentzian oscillators accounting for both the intraband and the interband contributions: $\varepsilon_c(\omega) = \varepsilon_\infty + \sum_j \frac{\omega_{p,j}^2}{\omega_{0,j}^2 - \omega^2 - i\gamma_j\omega}$. Here ε_∞ is the high-frequency dielectric constant, $\omega_{0,j}$, $\omega_{p,j}^2$ and γ_j are the center frequency, oscillator strength, and scattering rate of the j-th oscillator, respectively. The

model (red line in Fig. S5) agrees well with the experimental data and the fitting parameters are listed in Table. S1.

In Fig. S6 – Fig. S11, we show the gold disk antenna launching experiment with ZrSiSe crystals of varying thicknesses on top.

Section S4: Hyperbolic plasmon polariton dispersion with surface states.

The momentum of the hyperbolic plasmon polariton (HPP) modes with surface conductivity σ_{2D} obeys the following Fabry-Perot quantization condition:

$$q_l(\omega) = \frac{i\sqrt{\varepsilon_c}}{d\sqrt{\varepsilon_{ab}}}\left[\pi l + \arctan\left[i\frac{\varepsilon_0}{\varepsilon_1}\left(1-\frac{2q}{q_{2D}}\right)\right] + \arctan\left[i\frac{\varepsilon_2}{\varepsilon_1}\left(1-\frac{2q}{q_{2D}}\right)\right]\right] \qquad (S3)$$

where $l = 0, 1, 2$ is the mode index, d is the sample thickness, ε_0 (ε_2) is the dielectric function of the top (bottom) medium, and $\varepsilon_1 = \sqrt{\varepsilon_{ab}}\sqrt{\varepsilon_c}$ is the mean dielectric function of the hyperbolic material with the in-plane ($\varepsilon_{ab} < 0$) and out-of-plane ($\varepsilon_c > 0$) components having opposite signs. Here, q_{2D} is related to the (complex) surface state conductivity σ_{2D} of the hyperbolic metal via $\sigma_{2D} = \frac{\frac{i\omega(\varepsilon_0+\varepsilon_2)}{2}}{2\pi q_{2D}} = \frac{i\omega\kappa}{2\pi q_{2D}}$. Inside the hyperbolic regime of ZrSiSe, ε_1 is much larger than the dielectric function of the environment ($\varepsilon_0 = 1$, Air and $\varepsilon_2 = 1.94$, SiO_2). At $\omega = 6250\ cm^{-1}$ and considering only the real part, $\varepsilon_1 = \sqrt{\varepsilon_{ab}}\sqrt{\varepsilon_c} \approx 8.6i$ and therefore $i\frac{\varepsilon_j}{\varepsilon_1} \ll 1$ for $j = 0, 1$. Assuming $q_{2D} \ll q$, we can then approximate $\arctan\left[i\frac{\varepsilon_j}{\varepsilon_1}\left(1-\frac{2q}{q_{2D}}\right)\right] \approx i\frac{\varepsilon_j}{\varepsilon_1}\left(1-\frac{2q}{q_{2D}}\right)$ for $j = 0, 1$. Equation (S3) can then be simplified as:

$$q_l(\omega) = \frac{i\sqrt{\varepsilon_c}}{d\sqrt{\varepsilon_{ab}}}\left[\pi l + i\frac{\varepsilon_0+\varepsilon_2}{\sqrt{\varepsilon_{ab}}\sqrt{\varepsilon_c}}\left(1-\frac{2q}{q_{2D}}\right)\right] \qquad (S4)$$

and in the lossless limit (Re $\sigma_{2D} = 0$) we obtain:

$$q_l(\omega) = \frac{\pi l\sqrt{\varepsilon_c}\sqrt{|\varepsilon_{ab}|} + 2\kappa}{d|\varepsilon_{ab}| + 8\pi\ \mathrm{Im}(\sigma_{2D})/\omega} \qquad (S5)$$

The influence of the surface state metallicity will be parameterized by its complex sheet conductivity σ_{2D}. If the imaginary part of σ_{2D} at a given frequency is negative (positive), it will modify the HPP momentum to a larger (smaller) value, corresponding to enhanced (reduced) screening on the HPP dispersion.

To further illustrate the impact of surface state contribution on the HPP dispersion, we construct a toy model of the surface state dielectric function and evaluate the corresponding $\mathrm{Im}(r_p)$. As shown in Fig. S28 below, the band structure of a thin slab (5 layers) of ZrSiSe contains additional bands near the Fermi level compared to the bulk bands. These additional bands originated from the surface states of ZrSiSe and are consistent with reports in the literature for

ZrSiS/Se (see e.g. Ref. (*35*) for ZrSiSe and Refs. (*36*, *37*) for ZrSiS). Additional interband transitions associated with the surface states (e.g. green arrow in Fig. S28A) happen around $\omega_0 = 0.78$ eV ≈ 6290 cm^{-1} and will therefore influence the dispersion of the observed hyperbolic plasmons. We model the dielectric response of the surface state with surface plasma frequency ω_p^{SS} and a Lorentzian oscillator for the interband transition near ω_0. As shown in Fig. S28 panel B, with increasing surface plasma frequency from $\omega_p^{SS} = 1$ eV (green dotted line) to $\omega_p^{SS} = 2$ eV (green dashed line), the modeled plasmon dispersion gradually approaches the kink structure in the experimental data. The surface plasma frequency values used here (1 - 2 eV) are similar to those reported in the literature for ZrSiS (*36*).

Section S5: Angular and thickness dependence of hyperbolic plasmons near sample edges

In Fig. S12, we show the complete frequency-dependent near-field phase data (ϕ_4) for the 20 nm thin ZrSiSe crystal on the SiO_2/Si substrate. Dashed lines indicate the paths along which we extract the line profiles. The phase derivative line profile ($\frac{d\phi_4}{dr}$) and the corresponding simulation are shown in Fig. S13. Circles and triangles marks the real-space features that correspond to the principal (q_0) and higher-order (q_1) HPP modes. In Fig. S14 we also show the full near-field amplitude (S_4) and phase (ϕ_4) line profiles along the dashed lines in Fig. S12. The Fourier transform of the complex signal $S_4 e^{i\phi_4}$ are also shown in the right panel of Fig. S14 from $\omega = 5000$ cm^{-1} to 8333 cm^{-1}. Blue and orange symbols correspond to the principal (q_0) and higher-order (q_1) HPP modes and are consistent with the momenta extracted through line profile modeling in Fig. S13, as shown in the main text. The Fourier transform between 5000 cm^{-1} and 5780 cm^{-1} are also indicative another higher-order mode (q_2, green symbols) predicted by the Im(r_p) calculations.

The observed HPPs near sample edges are apparently angular dependent, as seen in Fig. S12 where the fringes in ϕ_4 are more pronounced on the right edge than at the left edge. Such angular dependence is contained within our quasistatic model (see Sec. S7) and stems from the polarization of the sample by the external field. This effect is indeed reflected in the modeled near-field image shown in Fig. S19. Importantly, the entire phase simulation image shown in Fig. S19 is generated with the polariton wavelengths obtained from the derivative line profile modelling in Fig. S13. The good agreement between the experiment and simulation on both edges of the crystal further confirms the accuracy of the extracted polariton wavelengths through line profile modelling.

As with the antenna launched HPPs, the tip-launched modes also show distinct thickness dependence that can be directly compared with the maximum of Im(r_p) calculated based on experimental dielectric functions (Fig. S1 and Fig. S5). In Fig. S15 we show the topography and corresponding near-field phase images ($\omega = 8333$ cm^{-1}) of a multi-terraced ZrSiSe crystal on

SiO_2/Si substrate. The thin flakes ranges from 24 to 122 nm in thickness and the phase linecut (Fig. S15C) shows an increase in fringe periodicities with increasing sample thickness. In particular, the distance between the first peak and the first dip (*t*) is approximately 0.13 times of modeled plasmon wavelength ($t \approx 0.13\lambda_p$). Such direct extraction of polariton wavelength has been utlized before in monolayer hBN (*33*) and serve as a quick estimate of the HPP wavelength in ZrSiSe. In the inset of Fig. S15C, we plot the extracted HPP momentum ($q_0 = \frac{2\pi}{\lambda_p}$) estimated based on the distance of the first peak and the first dip for various thicknesses of ZrSiSe. The data points are normalized to the momentum of the free-space light and agrees well with the calculated maxima of Im(r_p) (red curve).

Section S6: Modeling the near-field signal near antenna edges

To model the spatial profile of the signal near the edge of the gold disk, we develop an approximate solution for the scattered field created by a conducting disk, including the effects of diffraction. The basis of this approximation is Sommerfeld's solution to the famous problem of diffraction by a perfectly conducting screen (*51*). Below, we review this solution and use it to construct an approximate solution for a metallic disk covered by a thin optically hyperbolic film. Consider first a wave, incident at an angle α with respect to the plane with no component parallel to the edge of the conducting screen ($\beta = 0$), which we denote as the *y*-direction (Fig. S16 left). For concreteness, we first consider the case of a magnetic field $\boldsymbol{H} = H_y\hat{\boldsymbol{y}}$, where the scattered magnetic field has only one component along the *y*-direction, $H_y = U^{\perp}(x, z)$. The scattered magnetic field can be expressed through Fresnel diffraction integrals $F(z)$:

$$U^{\perp}(x, z; k) = U_0(x)\left(\frac{e^{ikz\sin\alpha + \frac{i\pi}{4}}}{\sqrt{\pi}}\left(F(\eta_+) + \frac{e^{-ikz\sin\alpha + \frac{i\pi}{4}}}{\sqrt{\pi}}F(\eta_-)\right) - isin(kz\sin\alpha)\right) \qquad (S6)$$

where $F(z) = \int_0^z e^{-i\kappa^2}d\kappa$, $\eta_\pm = \sqrt{2kr}\cos\frac{\phi\mp\alpha}{2}$, and $U_0(x) = E_0 e^{ikx\cos\alpha}$. Here *k* is the free-space photon wavevector, and (r, ϕ) represents polar coordinates in the xz-plane with $\tan\phi = \frac{z}{x}$ and $r = \sqrt{x^2 + z^2}$. For the other, orthogonal polarization with the incident electric field $\boldsymbol{E} = E_y\hat{\boldsymbol{y}}$, one obtains the second solution for $E_y = U^{\parallel}(x, z)$:

$$U^{\parallel}(x, z; k) = U_0(x)\left(\frac{e^{ikz\sin\alpha + \frac{i\pi}{4}}}{\sqrt{\pi}}\left(F(\eta_+) - \frac{e^{-ikz\sin\alpha + \frac{i\pi}{4}}}{\sqrt{\pi}}F(\eta_-)\right) - cos(kz\sin\alpha)\right) \qquad (S7)$$

An arbitrary incidence angle relative to the edge can be accomplished by introducing an angle β, understood as a latitude relative to the *y*-axis, shown in Fig. S16. The angles α, β are related to the incidence angles θ, ϕ of a spherical polar coordinate system by the relations:

$$\cos\alpha\cos\beta = \cos\psi\sin\theta$$
$$\sin\beta = \sin\psi\sin\theta$$
$$\sin\alpha\cos\beta = \cos\theta$$

The z-component of the scattered electric field for an incident *p*-polarized light can then be decomposed into the polarizations of the fundamental solutions $U^{\perp}, U^{\parallel}$ (52), yielding:

$$E_z^{sca}(x, y, z; k) = e^{ikysin\beta}\left[A\frac{i}{k}\frac{\partial}{\partial x}\left(U^{\perp}(x, z; k\cos\beta)\right) + B\frac{i\sin\beta}{k}\frac{\partial}{\partial z}\left(U^{\parallel}(x, z; k\cos\beta)\right)\right] \quad (S8)$$

The coefficients A, B arise from the decomposition of the polarization of the incident wave into components parallel and perpendicular to the edge of the screen and depend only on the polar and in-plane incidence angles α, β. We can then construct an approximate solution for a disk by solving for several angles ψ and plotting the diffraction pattern produced for each angle, with the out-of-plane component E_z plotted in Fig. S17a at a frequency of $\omega = 6600\ \text{cm}^{-1}$.

To check the validity of this approximation, we used the COMSOL package to simulate the scattered field distribution produced by a plane wave whose magnetic field was polarized parallel to the disk. This numerical approach was necessitated by the large free-space wavelength, which is comparable to the size of the metallic disk, invalidating the quasistatic approximation typically used in the modeling of the SNOM signal. The disk was included by implementing a perfectly conducting boundary condition on the surface of the disk inside of a physical domain of dimension 4 μm × 4 μm × 2 μm padded with perfectly matched layers of thickness 500 nm at each edge of the domain. A scattering boundary condition was implemented at the edge of the physical domain, and only the scattered field was extracted. The result of this simulation is plotted in Fig. S17b. The agreement between the approximation and the numerical solution is expected to hold only near the edge of the disk, which contains the crucial feature, namely a divergence of the field due to a sharp edge. The angular intensity distribution around the circumference of the disk is also captured by the approximate model, which can then be modified to account for the effect of the hyperbolic medium.

The introduction of the sample will bring with it the hyperbolic modes and modify the scattered field. The multiple branches of the polariton dispersion observed are derived by computing the poles in the reflection coefficient $r_p(q, \omega)$ in the absence of losses. In a realistic system with finite loss, the dispersion is instead dictated by the maxima in Im $r_p(q, \omega)$. We consider a three-layer system consisting of vacuum, sample and substrate, labeled as medium 0, 1 and 2, respectively. The divergence of $r_p(q, \omega)$ happens at a discrete set of values satisfying the condition:

$$2\pi l + \psi_{01} + \psi_{21} = 2k_1^z d \quad (S9)$$

for a medium of thickness d. The phase shifts ψ_{01}, ψ_{21} can be expressed in terms of reflection coefficients at the top and bottom interfaces, $r_{01} = e^{i\psi_{01}}$ and $r_{21} = e^{i\psi_{21}}$, respectively. The reflection coefficients r_{ij} at the interfaces are given by:

$$r_{ij}(q) = \frac{\frac{\varepsilon_j^{\perp}}{k_j^z} - \frac{\varepsilon_i^{\perp}}{k_i^z}}{\frac{\varepsilon_j^{\perp}}{k_j^z} + \frac{\varepsilon_i^{\perp}}{k_i^z}} \tag{S10}$$

where the z-component of the wavevector k_i^z of a p-polarized light in each medium is given by:

$$k_i^z(q) = \sqrt{\varepsilon_i^{\perp}}\sqrt{\frac{\omega^2}{c^2} - \frac{q^2}{\varepsilon_i^z}}, \qquad \mathrm{Im}\, k_i^z > 0 \tag{S11}$$

In the hyperbolic regime $(q)\omega/c)$, k_1^z is predominantly real, so the solutions of Eqn. (S9) are not confined to a surface but can exist within the bulk of the sample. A closed-form solution for the dispersion can only be obtained within the quasistatic approximation ($c \to \infty$). In that case, the reflection coefficients of Eqn. (S10) become independent of q and reduce to:

$$\beta_{ij} = \frac{\sqrt{\varepsilon_j^{\perp}}\sqrt{\varepsilon_j^z} - \sqrt{\varepsilon_i^{\perp}}\sqrt{\varepsilon_i^z}}{\sqrt{\varepsilon_j^{\perp}}\sqrt{\varepsilon_j^z} + \sqrt{\varepsilon_i^{\perp}}\sqrt{\varepsilon_i^z}} \tag{S12}$$

The original transcendental equation Eqn. (9) reduces to a linear equation for the dispersion of each mode q_l. In the particular case of launching by a conducting metallic edge, the observed fringes in real space can be understood as a beating between the various modes q_l in momentum space (*12*), giving a fringe spacing of:

$$\lambda_p = \frac{2\pi}{\Delta q_l} \approx -2id\frac{\sqrt{\varepsilon_1^{\perp}}}{\sqrt{\varepsilon_1^z}} \tag{S13}$$

with the last equality holding in the quasistatic limit.

Having previously obtained a solution for the field created in vacuum by a screen, this expression can be used as a building block to construct an approximate solution to the field produced by the system of the disk, sample, and substrate. Since the polariton wavelength (λ_p) is an order of magnitude smaller than the free-space photon wavelength λ_0, near the edge we expect a quasistatic approximation to be valid, permitting the use of an image method to introduce a sample (*12*). Using the field from Eqn. (S8), we introduce an equidistant series of images, as in the solution for the static field of a dielectric film between two media. We take the infinitely thin disk as the source of this static field, situated at the interface of media 1 and 2, that is, below the ZrSiSe layer. For an optically anisotropic material, the thickness of the film is further modified by the ratio of in-plane and z-axis dielectric function: $\frac{\sqrt{\varepsilon^t}}{\sqrt{\varepsilon^z}}$. The scattered near-field signal (S) can be approximated as the z-components of the field E_z^s obtained from the diffraction problem and the reflection coefficients β_{ij} from Eqn. (S12):

$$S(x,y,z) = (1-\beta_{01})E_z^{sca}(x,y,z+h) + \beta_{21}(1+\beta_{01})\sum_{n=1}^{\infty}\beta_{01}^n\beta_{21}^n E_z^{sca}\left(x,y,(2n+1)d\frac{\sqrt{\varepsilon^t}}{\sqrt{\varepsilon^z}}+h\right) \quad (S14)$$

To include the effects of demodulation, we compute the field at a discrete set of points above the sample:

$$h(t) = h_0 + \Delta h(1-\cos n\Omega t) \quad (S15)$$

to obtain the complex signal $\tilde{S_n} = S_n e^{i\phi_n}$. Here Ω is the tip-tapping frequency and we used tapping amplitude $\Delta h = 50$ nm and minimum position $h_0 = 5$ nm. The demodulated scattering amplitudes ($n = 3$) computed at a few different frequencies are shown in Fig. S18.

Section S7: Simulation of plasmonic fringes near the sample edge.

The input parameter into the model is a complex wavevector $Q = q_p(1+i\gamma)$, where $q_p = \frac{2\pi}{\lambda_p}$ and γ represents the dimensionless damping coefficient of the polariton mode. For very thin layers ($d \ll \lambda_p$), one can approximate the sample by a two-dimensional conducting layer with an effective sheet conductivity

$$\sigma_{eff} = \frac{i\omega\kappa}{2\pi Q}, \quad (S16)$$

with $\kappa = \frac{\varepsilon_0 + \varepsilon_2}{2}$ being the average permittivity of the surrounding media. The sample is modeled by a two-dimensional strip of width L at the boundary of two half-spaces with permittivities $\varepsilon_0 = 1$ (air) and $\varepsilon_2 = 1.94$ (SiO_2). The SNOM signal is taken to be proportional to the induced dipole moment on the probe. The scanning probe is modeled by a spheroid, with radius of curvature a = 40 nm and length L = 1400 nm. We compute the charge distribution n_i in the sample induced by the probe. This quantity can be found by combining Gauss's law with the charge continuity equation, giving

$$\Phi(\boldsymbol{r}) - V(\boldsymbol{r}) * n_i(\boldsymbol{r}) = \Phi_{ext}(\boldsymbol{r}), \quad (S17)$$

with the operation $A * B$ denoting convolution, $\Phi(\boldsymbol{r})$ being the full potential, and $V(\boldsymbol{r})$ being the in-plane Coulomb potential. The external field is taken to be constant with an incidence angle $\theta = 60°$ from the surface normal. Using the translation symmetry of the problem in the lateral direction, Eqn. (S17) is reduced to a one-dimensional integral equation. Replacing the derivatives by finite differences further simplifies Eqn. (S17) to a matrix inversion. The SNOM signal is then found by computing the dipole moment induced on the probe by the distribution $n_i(\boldsymbol{r})$. The complex signal (S) is calculated for a range of tip positions from each quantity $n_i(\boldsymbol{r})$ and then demodulated to the 4th harmonic in order to compare with the experimental line profiles shown in Fig. 3D in the main text and Fig. S13.

To simulate the near-field phase-contrast of triangular-shaped samples (Fig. S19), we created the two-dimensional image by solving Eqn. (S17) for a semi-infinite conducting sheet near a sample

edge. We obtained two different solutions for the two cases of the in-plane component of the external field being parallel and anti-parallel to the sample edge. These two solutions were used for each edge of the triangular flake separately, and the intermediate region was interpolated between the edges. This approach is valid provided that the width of the sample at that point $L \gg \mathrm{Im}\, Q$, see Eqn. (S16).

Section S8: Survey of electronic loss in plasmonic and excitonic hyperbolic materials

In this section, we list the reflectance, dielectric function and optical conductivities ($\sigma(\omega) = \sigma_1 + i\sigma_2$) of various plasmonic and excitonic hyperbolic materials reported in the literature. To quantify the electronic loss, the ratio $\frac{\sigma_2}{\sigma_1}$ is calculated based on the reported or extracted optical conductivities.

Section S9: Electronic band structure calculations of ZrSiSe

The electronic structure of the system was investigated with density functional theory (DFT). DFT calculations were carried out at the level of DFT plus onsite Hubbard U and intersite V (DFT+U+V) (*53*), as implemented in the Octopus code (*54*), which delivers an hybrid-like quality of the band structure at a fraction of the computational cost (*55*). Experimental lattice constants of $a = 3.623$ Å and $c = 8.365$ Å were employed. For the slab configuration, containing 5 layers of ZrSiSe, a 16 Å vacuum region was chosen to properly converge the bands along the non-periodic dimension z. The ground state was calculated by discretizing the equations in real-space with a spacing of 0.159 Å and spin-orbit coupling was fully accounted for valence electrons while core electrons were treated with relativistic HGH pseudopotentials (*56*). The Brillouin zone was sampled with a 16×16×8 Monkhorst-Pack grid for the bulk and a 15×15 grid for the slab geometries.

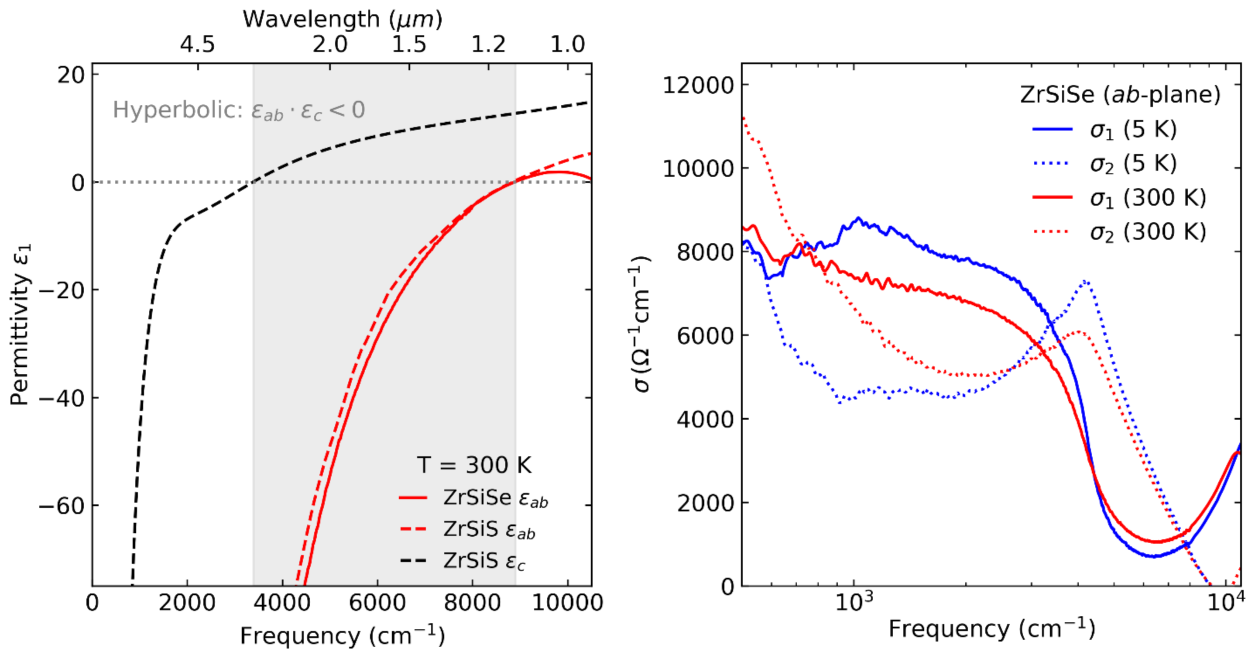

Fig. S1. Optical conductivities of ZrSiS and ZrSiSe. (Left) Real part of the dielectric function of ZrSiS (dashed lines) and ZrSiSe (solid line). The gray-shaded region indicates the frequency range where ZrSiS is hyperbolic. (Right) *ab*-plane optical conductivities of ZrSiSe at 300 K and 5 K. Solid and dotted lines represent the real and imaginary parts of $\boldsymbol{\sigma(\omega) = \sigma_1 + i\sigma_2}$, respectively.

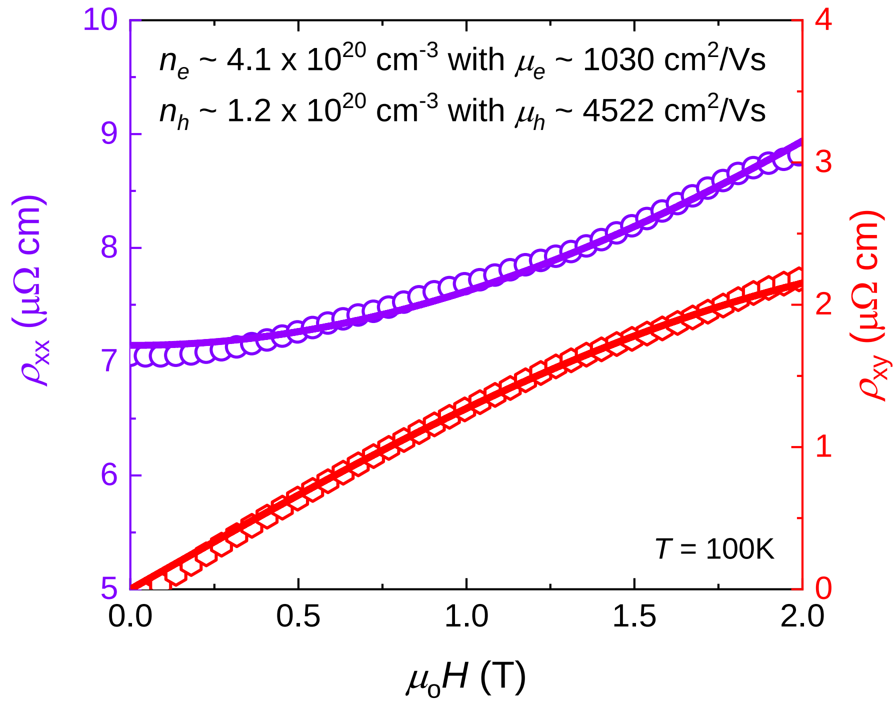

Fig. S2. **Magnetotransport measurements and fitting.** Two-band model fits of longitudinal (ρ_{xx}) and transverse (ρ_{xy}) resistivity for ZrSiSe at 100 K.

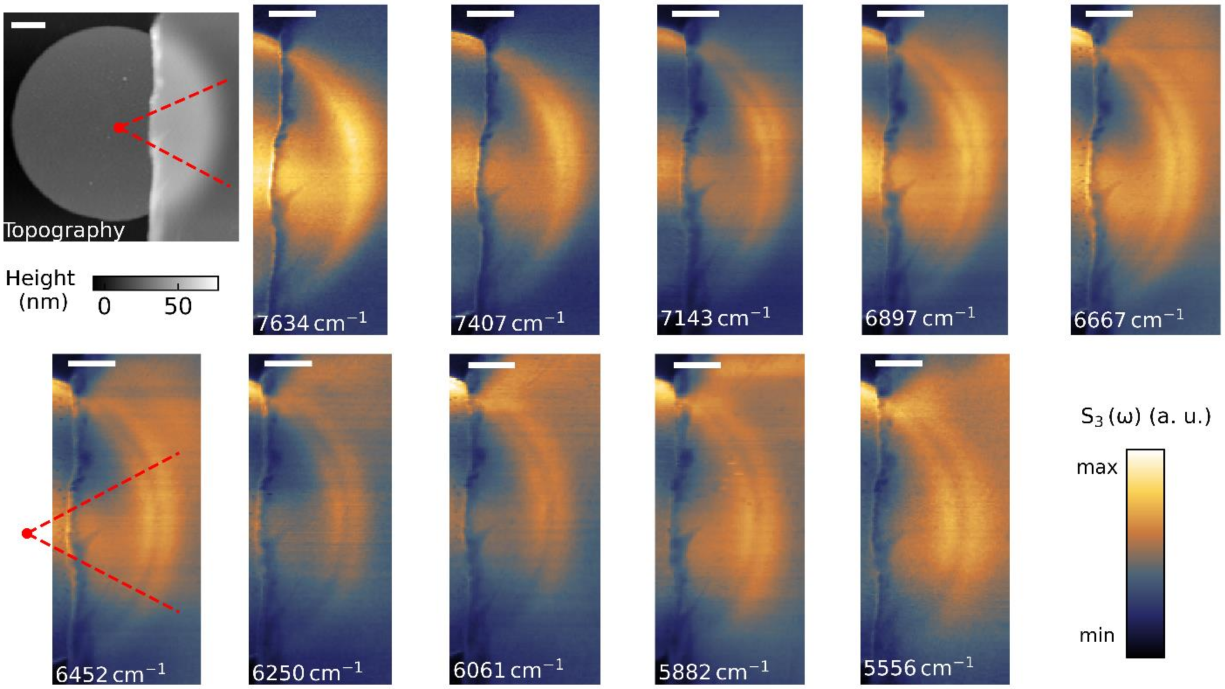

Fig. S3. **Frequency dependent near-field data.** Topography and frequency-dependent near-field scattering amplitude data (S_3) of the 26 nm ZrSiSe sample on a gold disk antenna. Scale bars in all panels are 300 nm. Red dashed lines indicate the sector region used to average the line profiles of S_3 along the perimeter of the disk antenna and are kept the same for all frequencies.

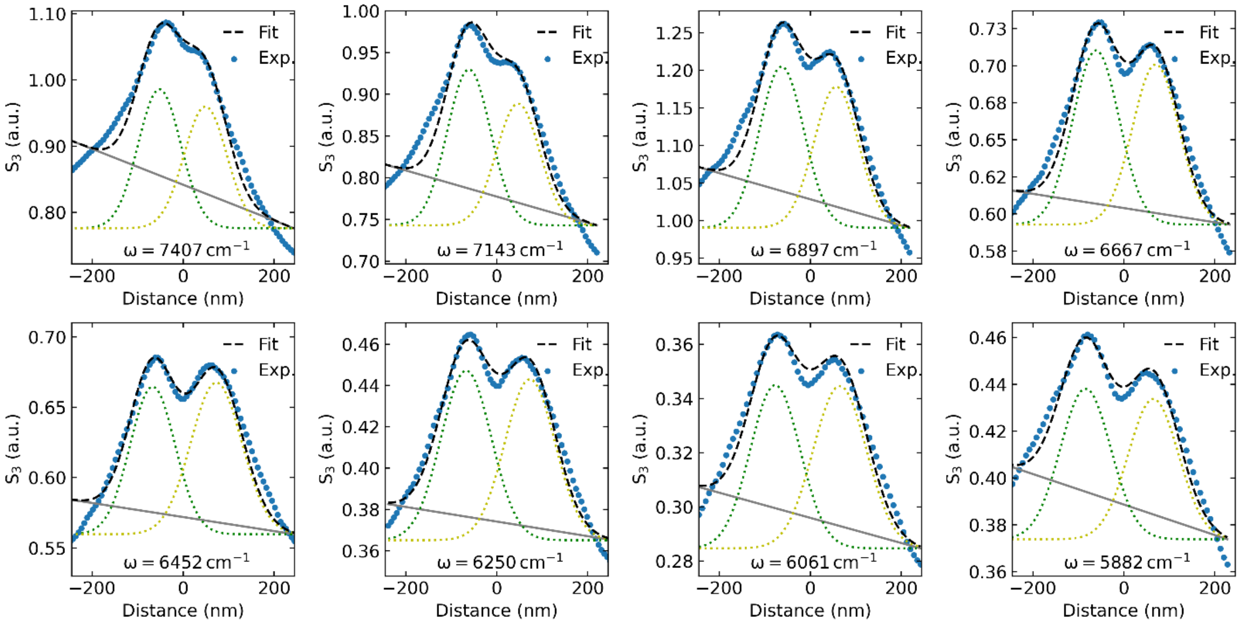

Fig. S4. **Extracted line profiles and fitting.** Frequency-dependent line profiles in the sample region from Fig.S3 are shown as blue dots. The extracted line profiles are fitted with two Gaussian profiles (green and yellow dotted lines) and linear backgrounds (gray solid lines). Black dashed lines are the sum of the Gaussians and the background, showing good agreement with the experiment.

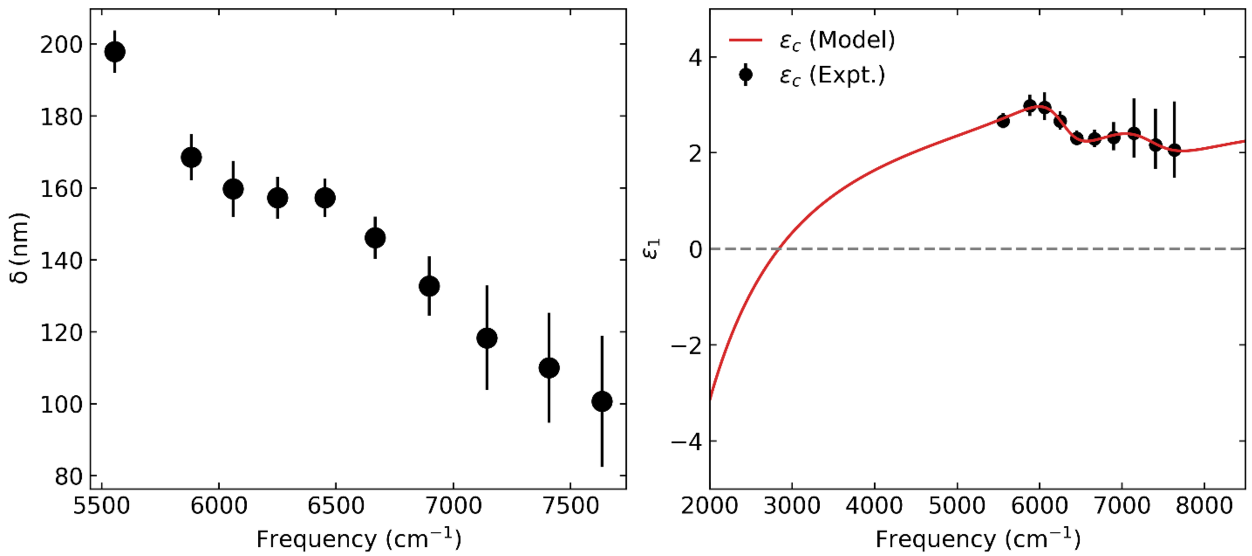

Fig. S5. Double-ring separation and Drude-Lorentz model of the *c*-axis dielectric function for ZrSiSe. (Left) Experimental peak separation $\delta(\omega)$ obtained from the fitting in Fig. S4 and the slope correction discussed in the Materials and Methods section. (Right) Drude-Lorentz model fitting of the *c*-axis dielectric function data (black dots), obtained through the antenna launching experiment.

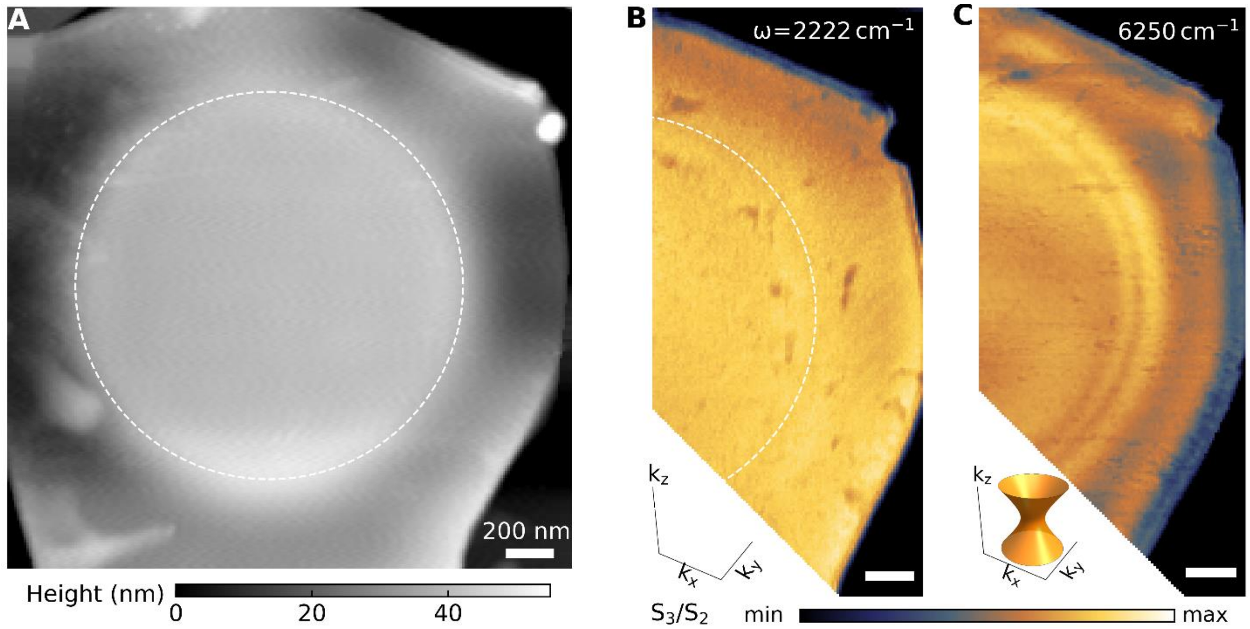

Fig. S6. Hyperbolic ray launching experiment for fully covered Au antenna.
(**A**), Topography image of a 28 nm ZrSiSe crystal on top of a 2 μm wide Au circular antenna. The normalized near-field amplitudes ($\frac{S_3}{S_2}$) outside ($\omega = 2222\ \mathrm{cm}^{-1}$) and inside ($\omega = 6250\ \mathrm{cm}^{-1}$) the hyperbolic regime are shown in panel (**B**) and (**C**), respectively. Insets in panels **B** and **C** are the schematic of the isofrequency surfaces. Dashed lines in **A** and **B** indicate the boundary of the underlying Au antenna. Scale bars in all panels are 200 nm.

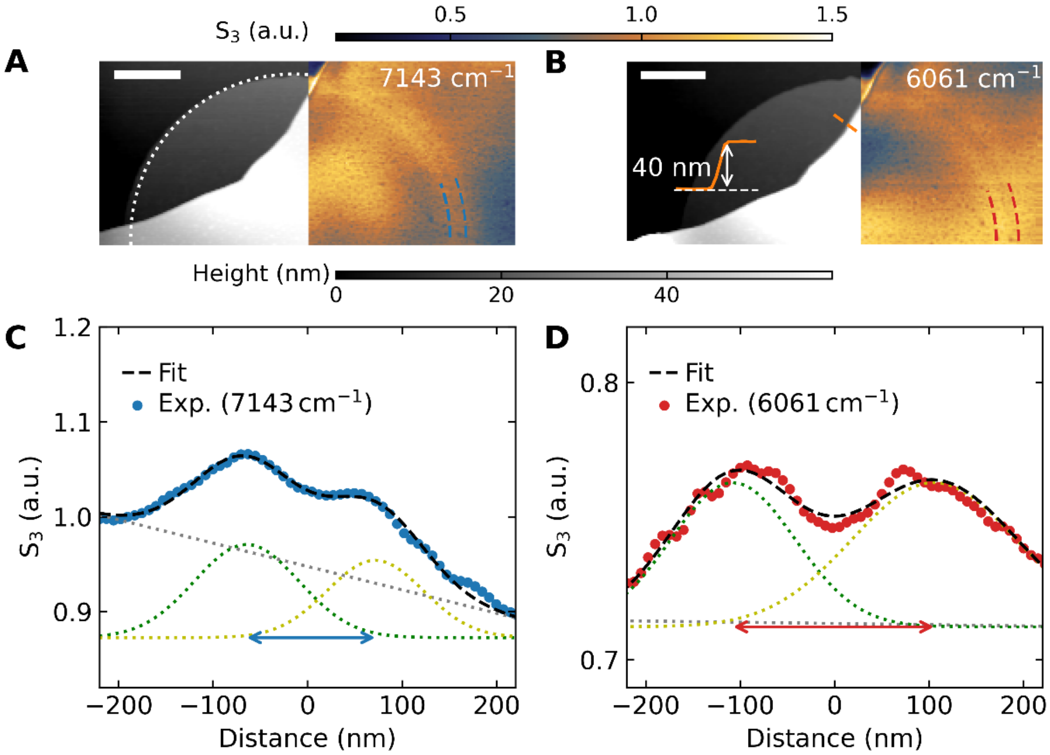

Fig. S7. Hyperbolic ray launching data and fitting of a 40 nm ZrSiSe crystal on circular gold antenna. Topography (left) and the near-field amplitude data (right) collected at (**A**) $\omega = 7143\ \mathrm{cm}^{-1}$ and (**B**) $\omega = 6061\ \mathrm{cm}^{-1}$. The white dotted line indicates the boundary of gold. Blue and red dashed lines indicate the double-ring features at $7143\ \mathrm{cm}^{-1}$ and $6061\ \mathrm{cm}^{-1}$, respectively. Inset in **B** is a topography linecut along the orange dashed line. Scalebars are 500 nm. The averaged line profiles of the near-field amplitude along the perimeter of the circular antenna are shown for (**C**) $\omega = 7143\ \mathrm{cm}^{-1}$ and (**D**) $\omega = 6061\ \mathrm{cm}^{-1}$. The line profiles are fitted with two Gaussian profiles (green and yellow dotted lines) and a linear background (gray dotted line).

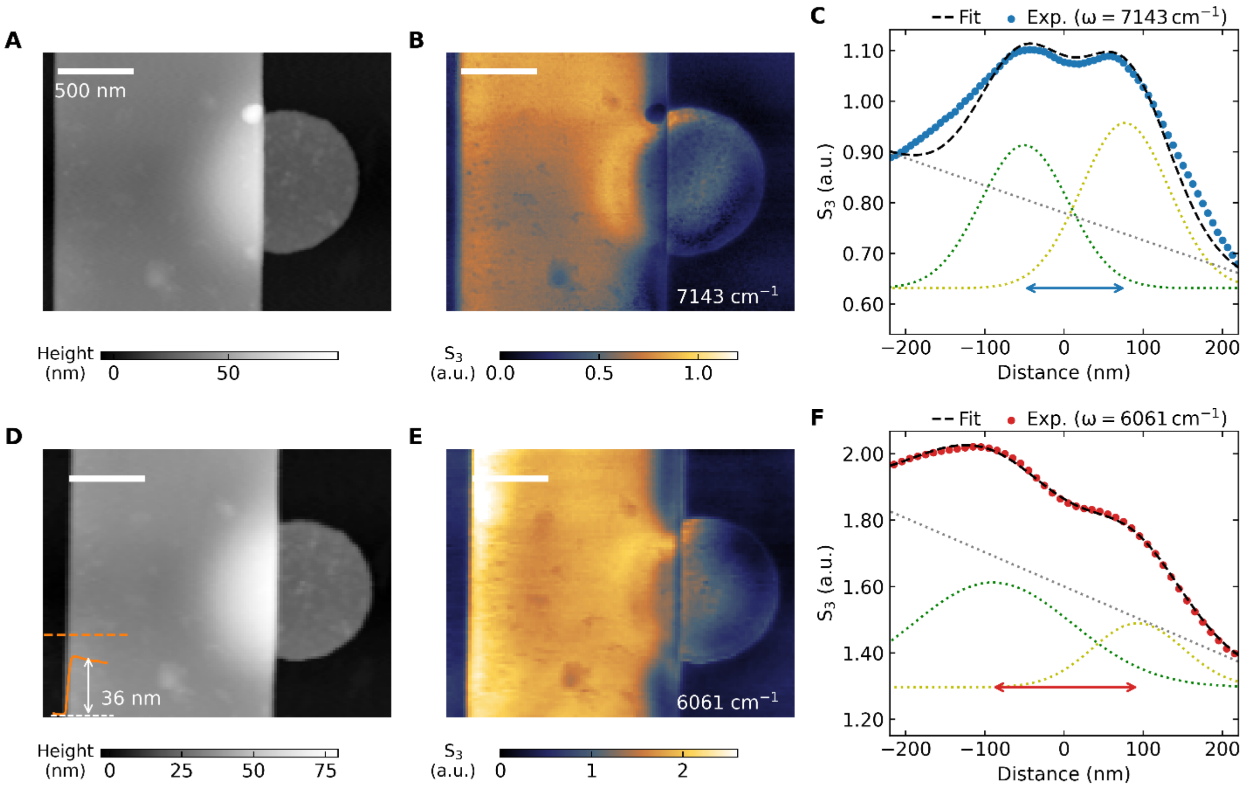

Fig. S8. Hyperbolic ray launching data and fitting of a 36 nm ZrSiSe crystal on circular gold antenna. Topography and the corresponding near-field amplitude data (S_3) at (**A**, **B**) $\omega = 7143\ \mathrm{cm}^{-1}$ and (**D**, **E**) $\omega = 6061\ \mathrm{cm}^{-1}$. Inset in panel **D** is the topography line profile along the orange dashed line. The extracted near-field amplitude line profiles on the sample along the perimeter of the antenna are shown for $7143\ \mathrm{cm}^{-1}$ and $6061\ \mathrm{cm}^{-1}$ in **C** and **F**, respectively. The line profiles are fitted with Gaussian functions (green and yellow dotted lines) and a linear background (grey dotted line). Scale bars in panels **A-D** are 500 nm.

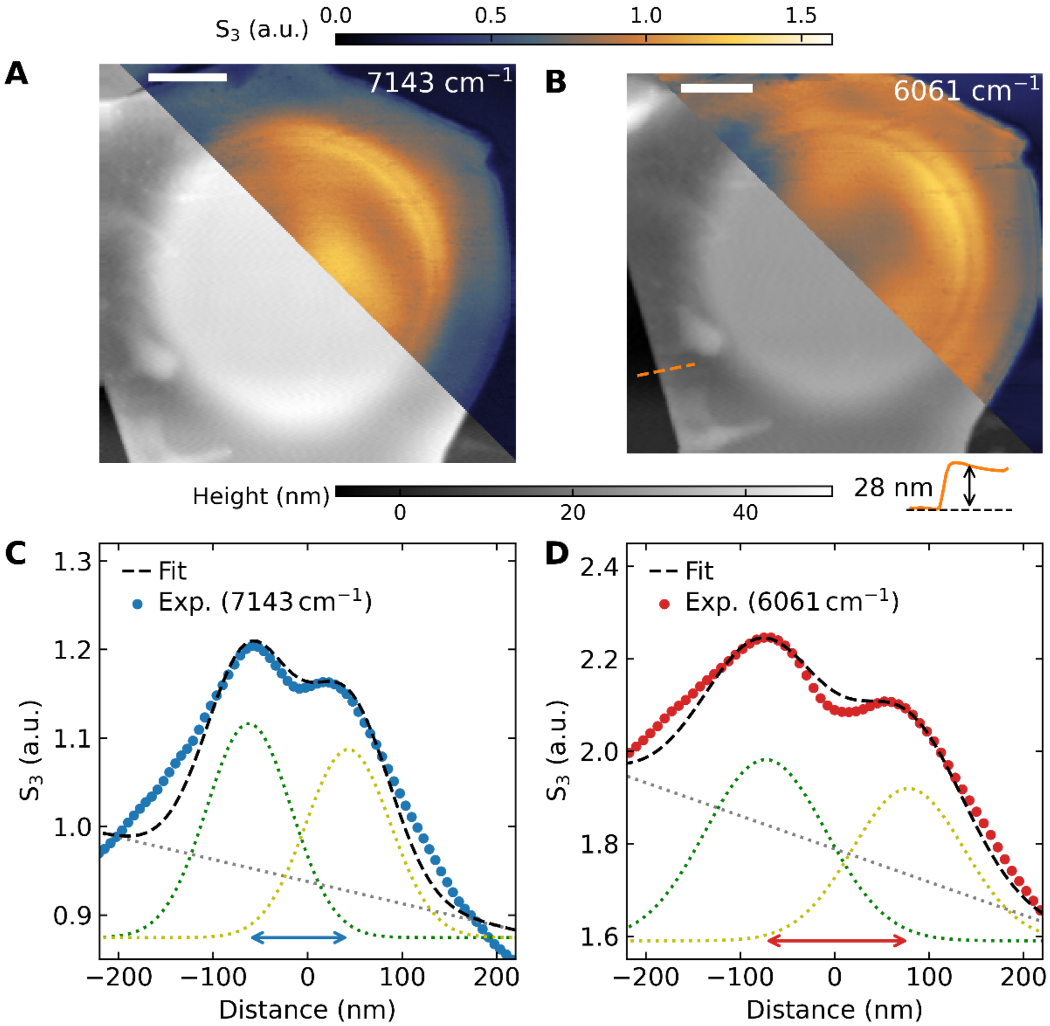

Fig. S9. Hyperbolic ray launching data and fitting of a 28 nm ZrSiSe crystal on circular gold antenna. Topography (bottom) and the near-field amplitude data (top) at (**A**) $\omega = 7143\ \mathrm{cm}^{-1}$ and (**B**) $\omega = 6061\ \mathrm{cm}^{-1}$. The extracted near-field amplitude line profiles on the sample along the perimeter of the antenna are shown for $7143\ \mathrm{cm}^{-1}$ and $6061\ \mathrm{cm}^{-1}$ in **C** and **D**, respectively. The line profiles are fitted with Gaussian functions (green and yellow dotted lines) and a linear background (grey dotted line). Scale bars in panels **A**,**B** are 500 nm.

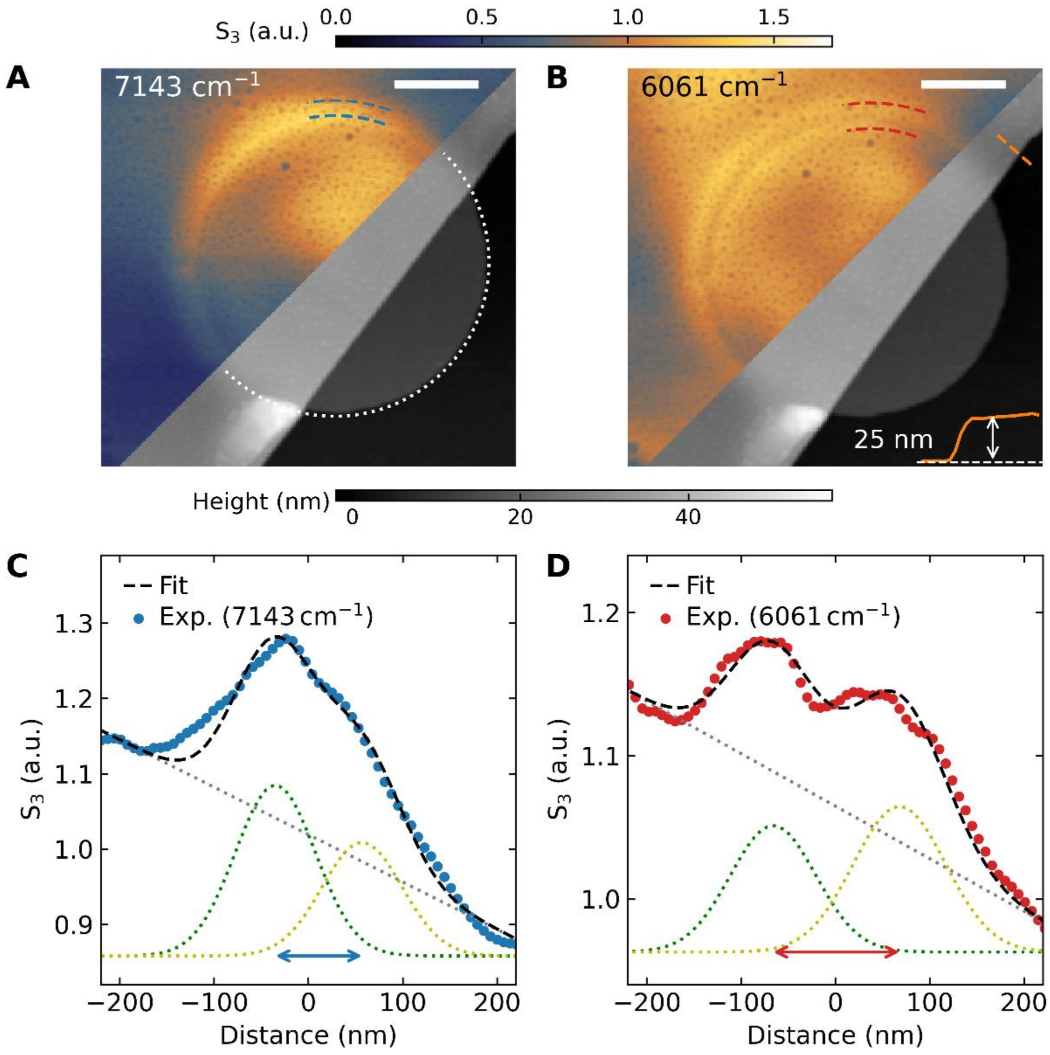

Fig. S10. Hyperbolic ray launching data and fitting of a 25 nm ZrSiSe crystal on circular gold antenna. Topography (bottom) and the near-field amplitude data (top) collected at (**A**) $\omega = 7143\ \mathrm{cm}^{-1}$ and (**B**) $\omega = 6061\ \mathrm{cm}^{-1}$. The white dotted line indicates the boundary of gold. Blue and red dashed lines indicate the double-ring features at $7143\ \mathrm{cm}^{-1}$ and $6061\ \mathrm{cm}^{-1}$, respectively. Inset in **B** is a topography linecut along the orange dashed line. Scalebars are 500 nm. The averaged line profiles of the near-field amplitude along the perimeter of the circular antenna are shown for (**C**) $\omega = 7143\ \mathrm{cm}^{-1}$ and (**D**) $\omega = 6061\ \mathrm{cm}^{-1}$. The line profiles are fitted with two Gaussian profiles (green and yellow dotted lines) and a linear background (gray dotted line).

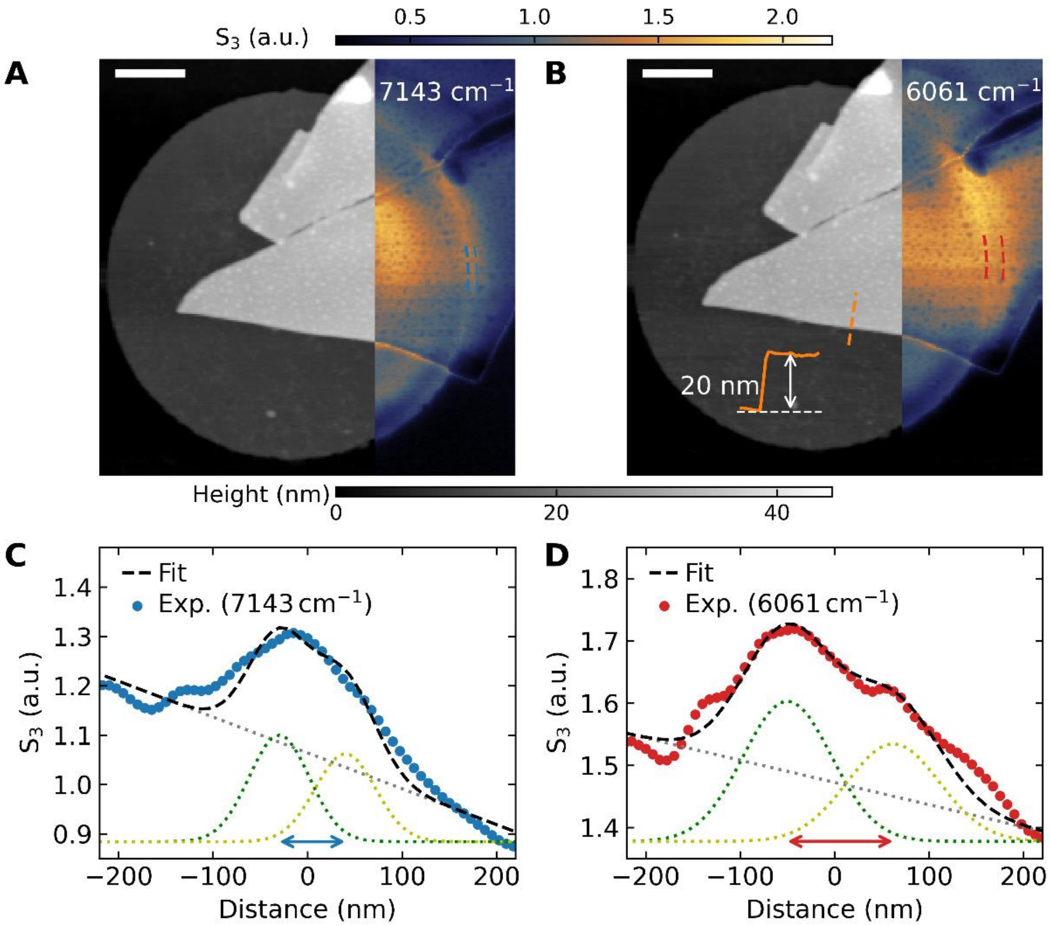

Fig. S11. Hyperbolic ray launching data and fitting of a 20 nm ZrSiSe crystal on circular gold antenna. Topography (left) and the corresponding near-field amplitude data (right) at (**A**) $\omega = 7143\ \mathrm{cm}^{-1}$ and (**B**) $\omega = 6061\ \mathrm{cm}^{-1}$. Inset in panel **B** is the topography line profile along the orange dashed line. The extracted near-field amplitude line profiles on the sample along the perimeter of the antenna are shown for $7143\ \mathrm{cm}^{-1}$ and $6061\ \mathrm{cm}^{-1}$ in **C** and **D**, respectively. The line profiles are fitted with Gaussian functions (green and yellow dotted lines) and a linear background (grey dotted line). Scale bars in **A**,**B** are 500 nm.

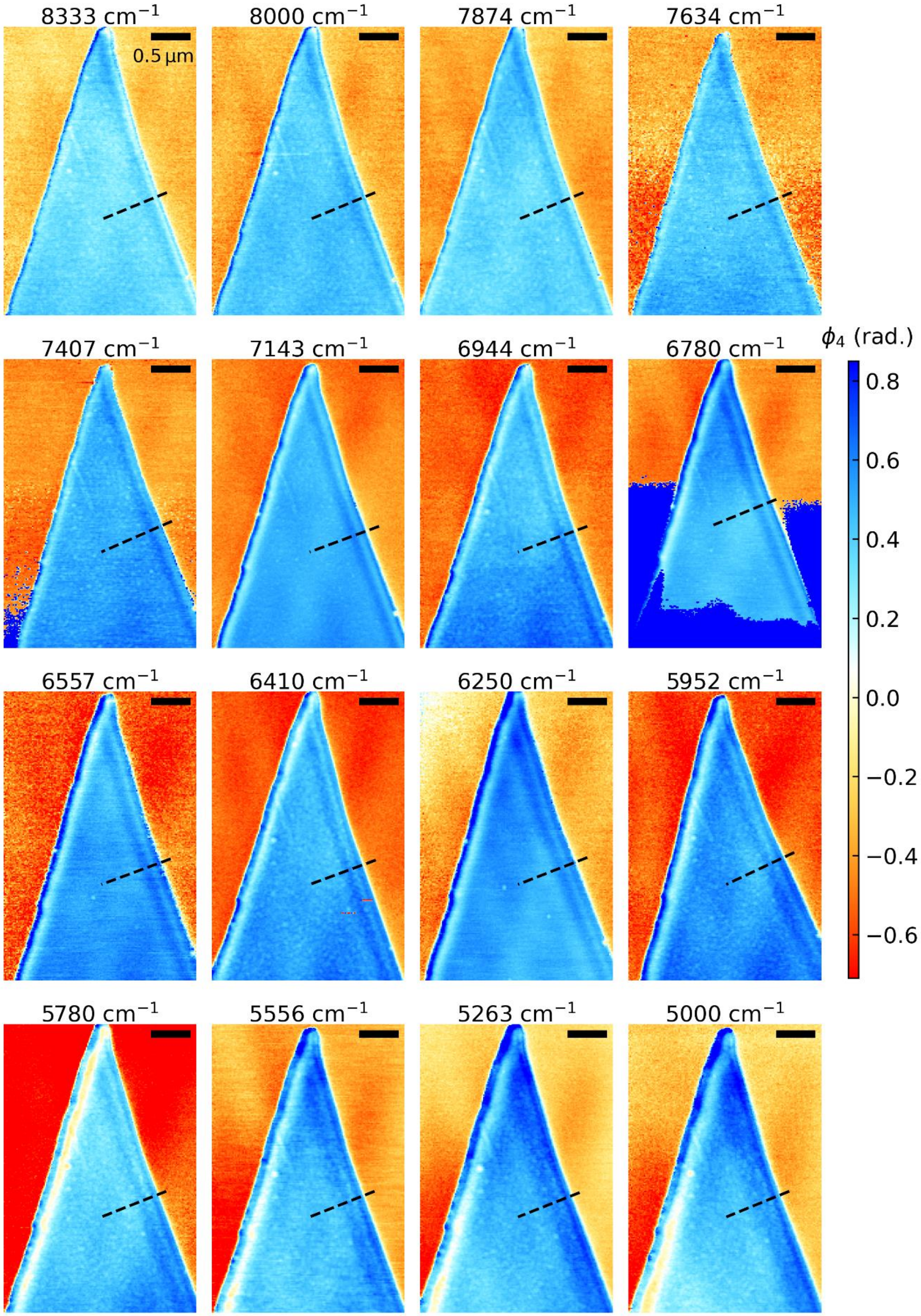

Fig. S12. Edge launching near-field imaging data. Frequency-dependent ($\omega = 8333 - 5000$ cm^{-1}) near-field phase (ϕ_4) for the 20 nm ZrSiSe on SiO_2/Si. Scale bars are 500 nm.

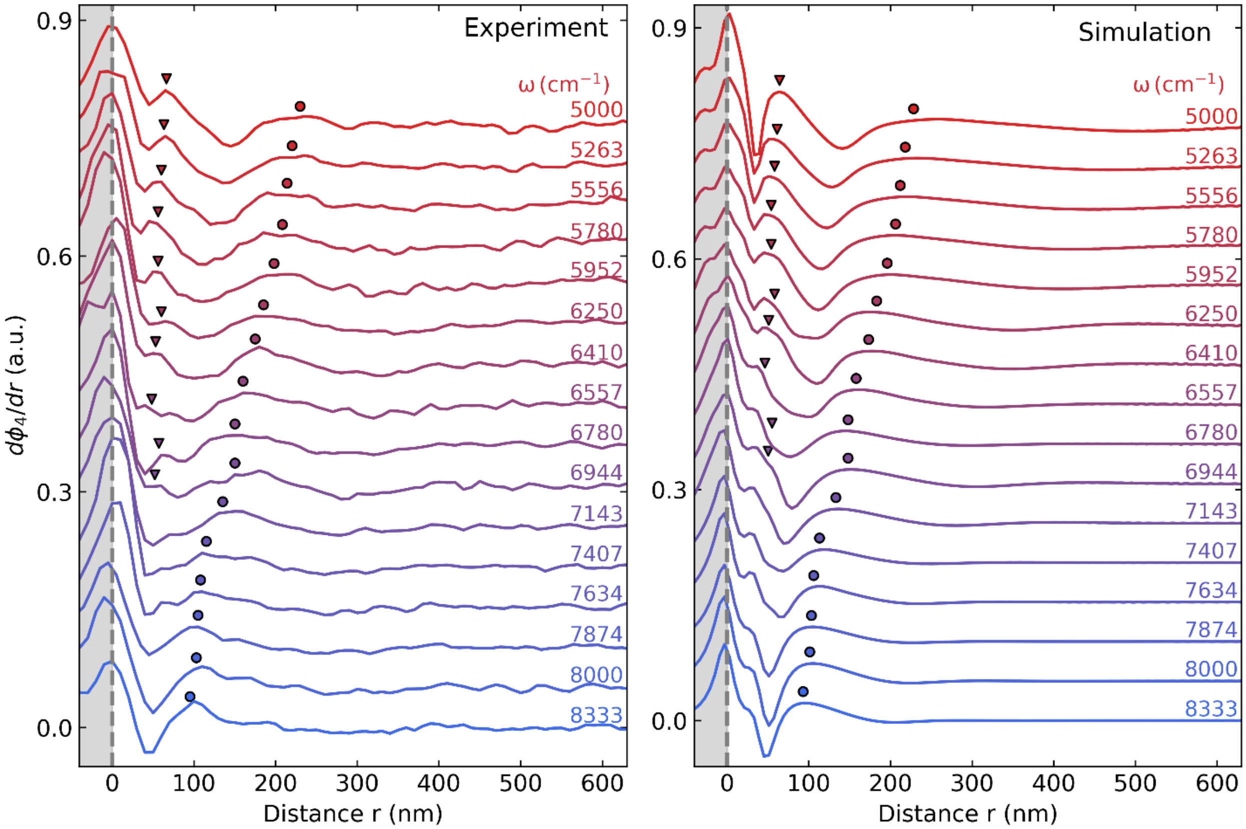

Fig. S13. Experimental and simulated phase derivative line profiles near edges of ZrSiSe. (Left) Line profiles of near-field phase derivative ($\frac{d\phi_4}{dr}$) along the black dashed lines in Fig. S12 for the 20 nm ZrSiSe on SiO_2/Si substrate. (Right) Simulation of the phase derivative line profiles at corresponding frequencies. Colored circles and triangles mark the positions of the principal (q_0) and higher-order (q_1) hyperbolic plasmon polaritons, respectively.

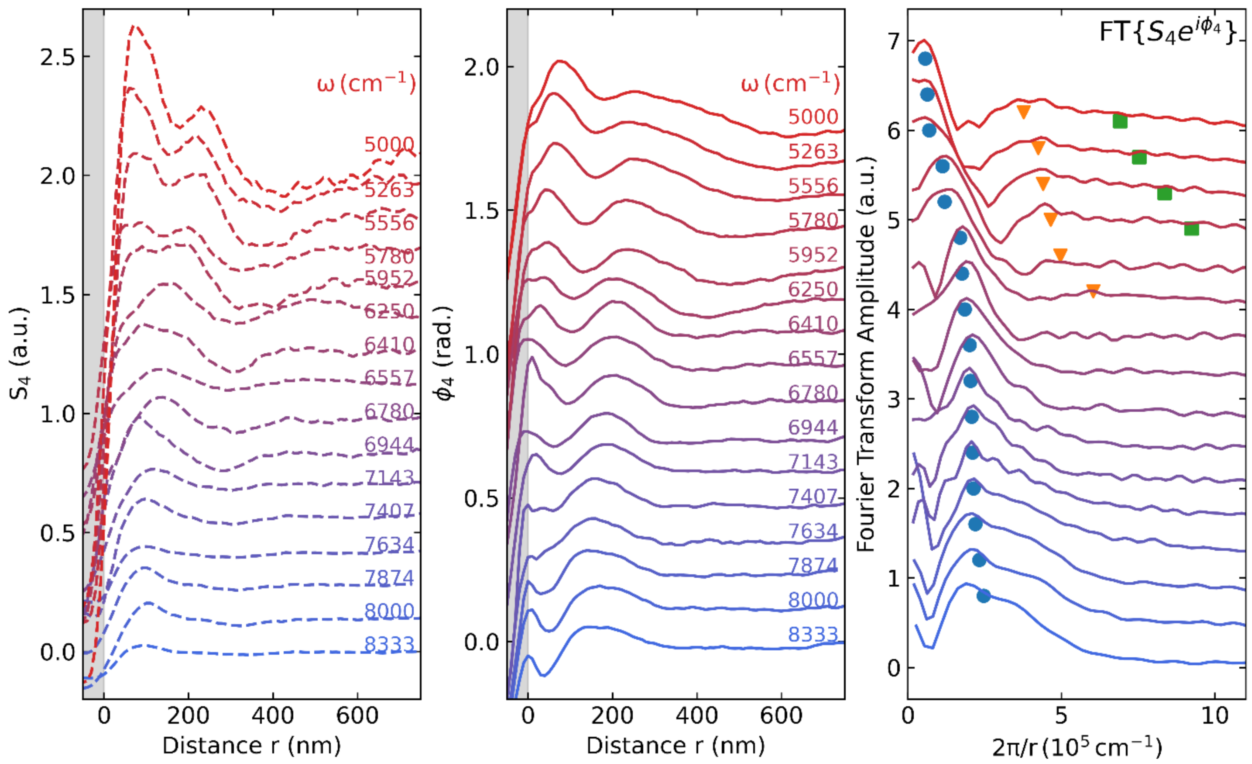

Fig. S14. Amplitude and phase line profiles near edges of ZrSiSe and the corresponding Fourier transform amplitude. (Left and Middle) Experimental line profiles of near-field amplitude (S_4) and phase (ϕ_4) along the black dashed lines in Fig. S12 for the 20 nm ZrSiSe on SiO_2/Si. (Right) Fourier transform of the complex signal $S_4 e^{i\phi_4}$ from $\omega = 5000\ \text{cm}^{-1}$ (red) to $8333\ \text{cm}^{-1}$ (blue). Blue, orange and green symbols represent the mometa of the principal (q_0) and higher-order hyperbolic plasmon polaritons modes (q_1, q_2), respectively.

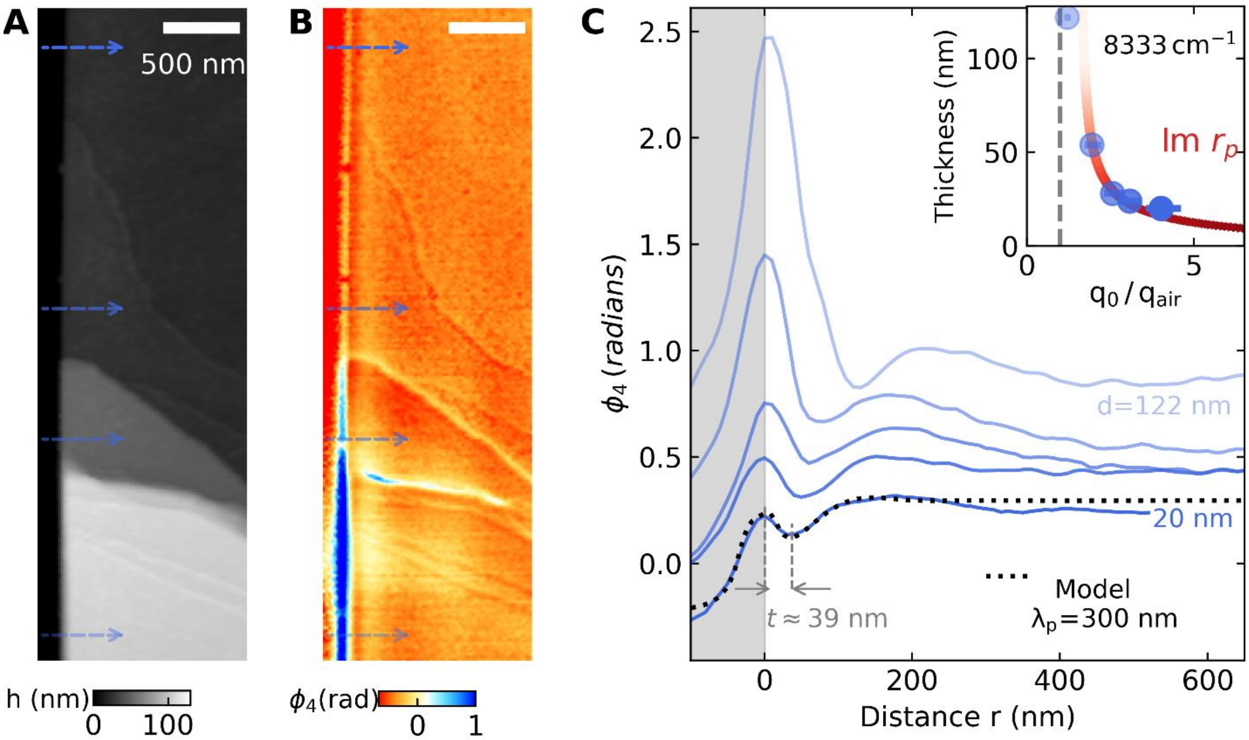

Fig. S15. Thickness dependence of edge launched HPP thin ZrSiSe. (**A**), Topography of a multi-terraced ZrSiSe crystal on SiO_2/Si substrate. (**B**), Near-field phase image (ϕ_4) in the same region taken at $\omega = 8333\ \mathrm{cm}^{-1}$. The four arrows from top to down corresponds to thickness of 24 nm, 28 nm, 50 nm and 122 nm, respectively. (**C**), Phase line profiles at various thicknesses along the arrow positions in panel (B), the line profile and model for 20 nm ZrSiSe are taken from Fig. S12 (right edge). Gray shaded region indicates the substrate. Black dotted line indicate the modeled line profile with plasmon wavelength (λ_p) of 300 nm. Inset depicts the thickness dependence of extracted (principal) HPP momentum $q_0 = \frac{2\pi}{\lambda_p}$ normalized by the momentum of free-space light ($q_{air} = \frac{2\pi}{\lambda}$), showing good agreement with the calculated Im (r_p) (red curve) based on experimental dielectric constants of ZrSiSe.

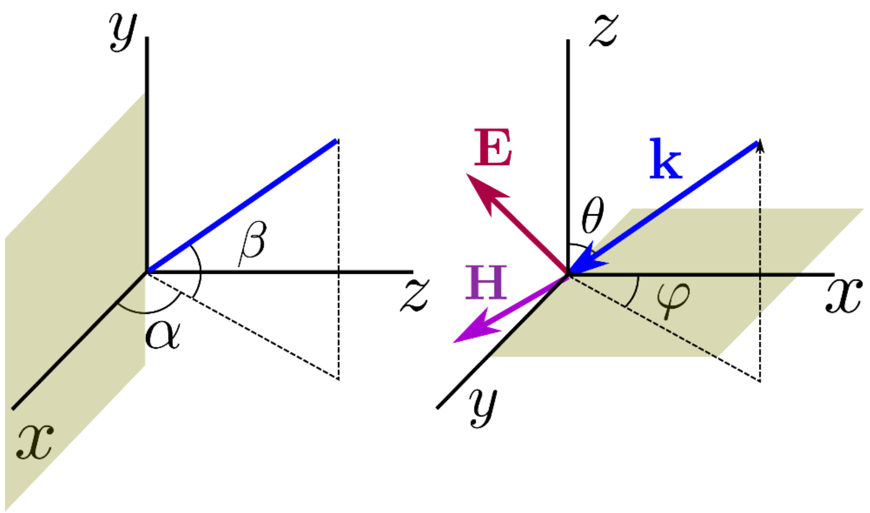

Fig. S16. Simulation coordinates. A schematic illustrating different choices of coordinate systems used in the expressions for the scattered fields. The shaded region represents the conducting screen creating the diffraction pattern, with the screen running parallel to the *y*-axis.

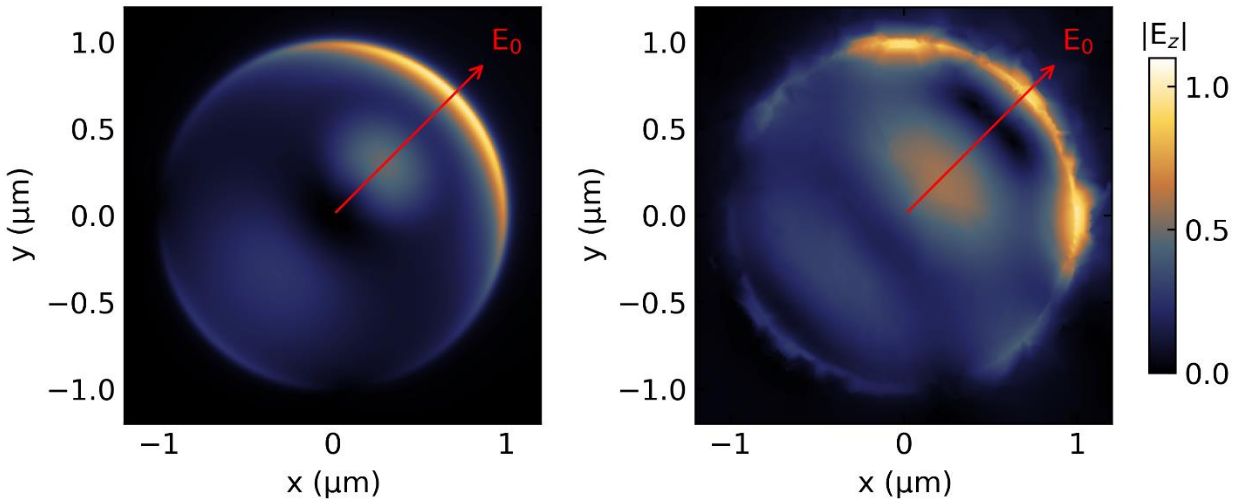

Fig. S17. Simulation of z-axis electric field of a bare Au disk antenna. Absolute value of the z-component of the scattered field E_z at a height of 25 nm above the disk, obtained using the approximate model (Left) and the numerical solution (Right). The red arrow in both panels highlights the direction of the incident field.

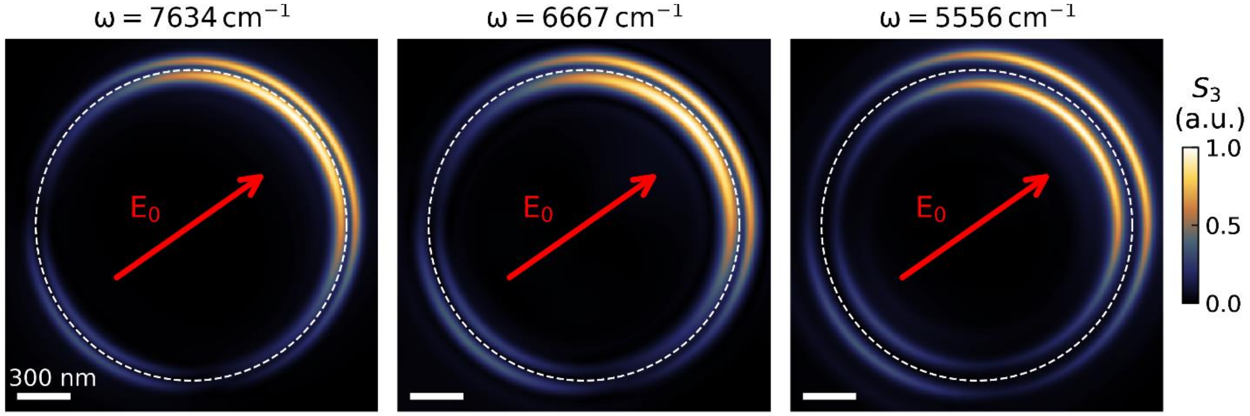

Fig. S18. Hyperbolic polariton antenna launching simulation. Simulated near-field amplitude of ZrSiSe (26.5 nm) on a gold disk (25 nm) obtained from the approximate model at $\omega = 7634\ \mathrm{cm}^{-1}, 6667\ \mathrm{cm}^{-1}, 5556\ \mathrm{cm}^{-1}$. The white dashed line shows the edge of the gold disk and the red arrow indicates the direction of the field.

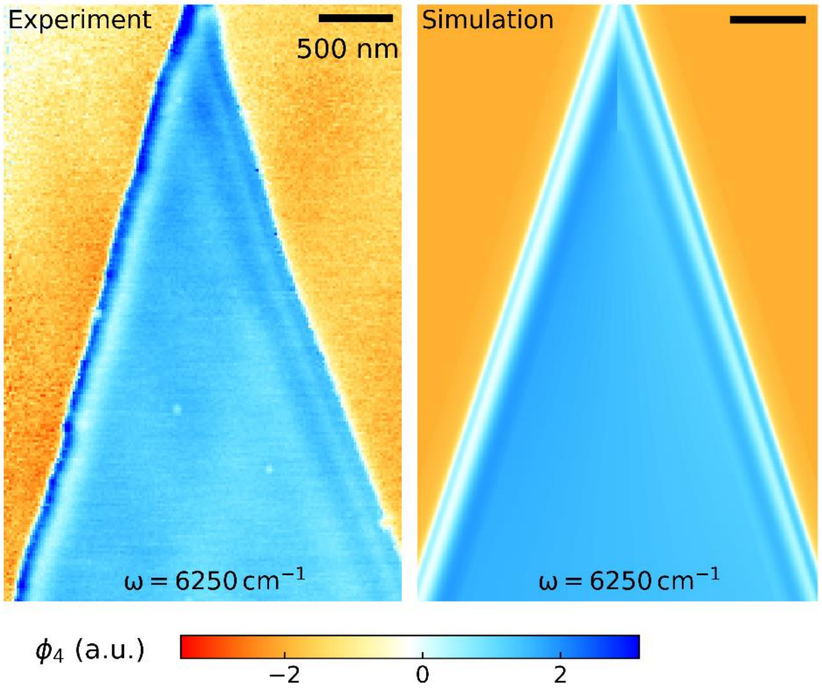

Fig. S19. Angular dependence in triangular-shaped thin crystal of ZrSiSe.
(Left) Experimental phase-contrast image (ϕ_4) of hyperbolic plasmon polaritons near the edges of the crystal at $\omega = 6250\ \mathrm{cm}^{-1}$, showing apparent differences in fringe spacing for two edges of the flake. (Right) Simulation of the phase contrast ϕ_4 image using the same polartion wavelengths: $\lambda_{p0} = 580\ nm$ and $\lambda_{p1} = 140\ nm$ for the principal (q_0) and higher-order (q_1) mode, respectively.

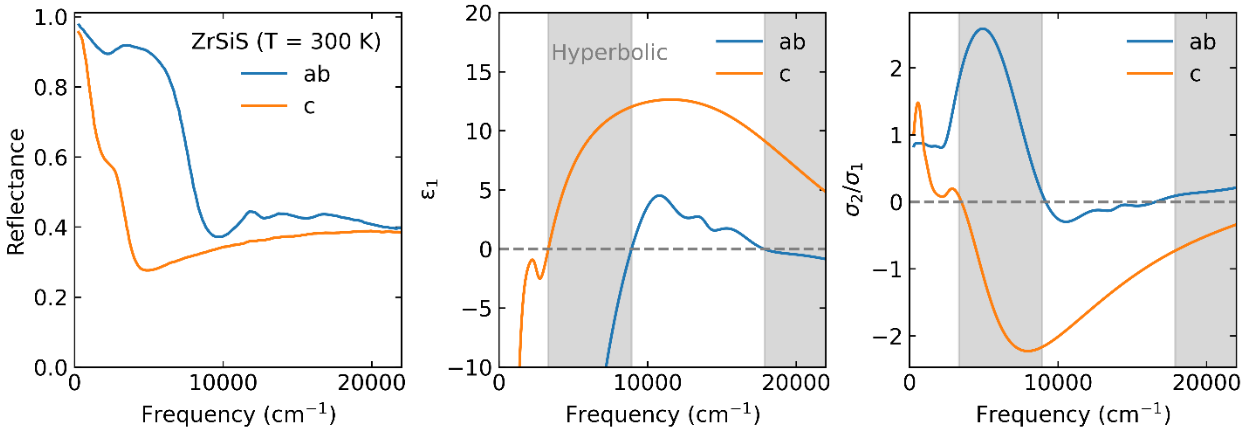

Fig. S20. ZrSiS. Anisotropic reflectance, real part of the dielectric function and $\frac{\sigma_2}{\sigma_1}$ ratio of ZrSiS (*25*). Gray shaded regions indicate the hyperbolic frequency regime.

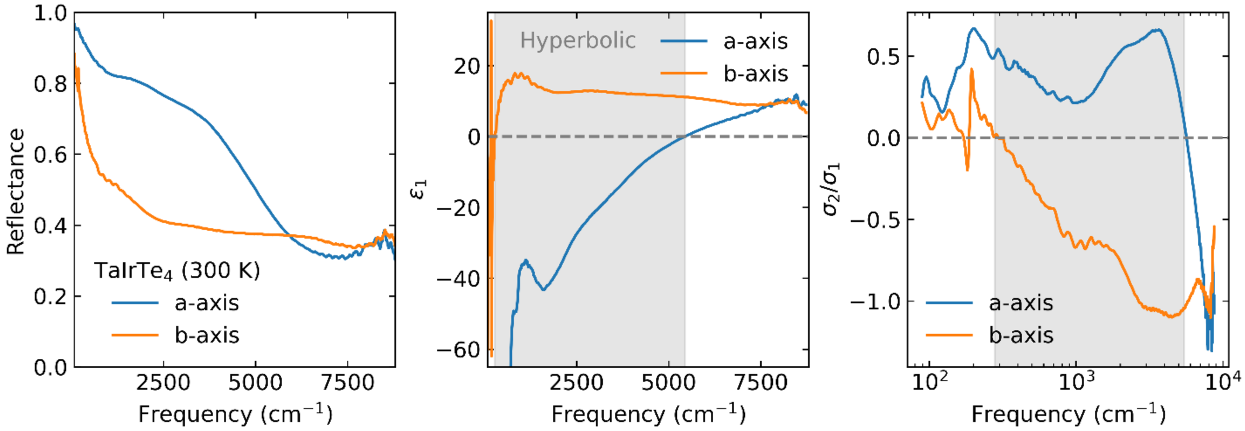

Fig. S21. TaIrTe$_4$. Anisotropic reflectance, real part of the dielectric function and $\frac{\sigma_2}{\sigma_1}$ ratio of TaIrTe$_4$ (*24*).

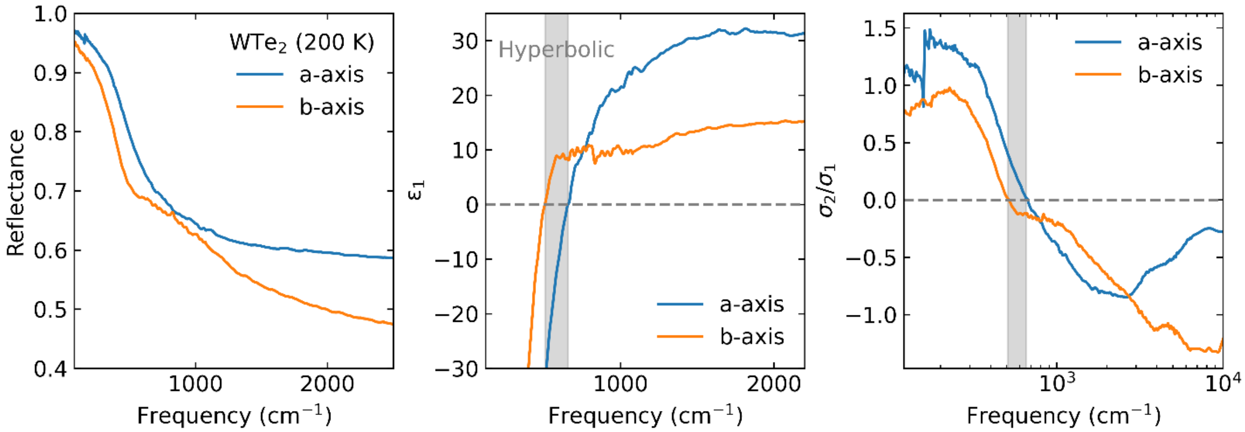

Fig. S22. WTe$_2$. Anisotropic reflectance, real part of the dielectric function and $\frac{\sigma_2}{\sigma_1}$ ratio of WTe$_2$ (*22*).

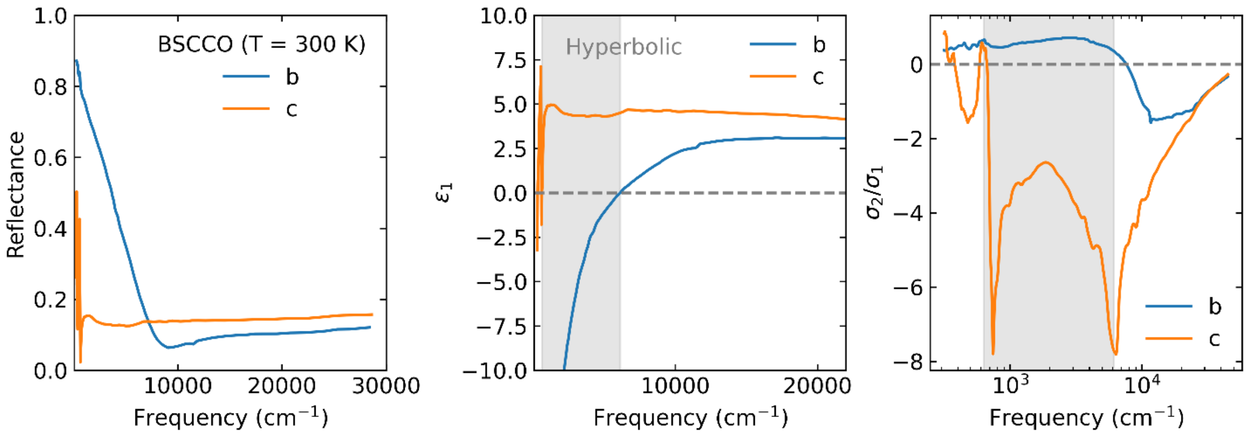

Fig. S23. $Bi_2Sr_2CaCu_2O_8$. Anisotropic reflectance, real part of the dielectric function and $\frac{\sigma_2}{\sigma_1}$ ratio of $Bi_2Sr_2CaCu_2O_8$ (BSCCO) (*38*).

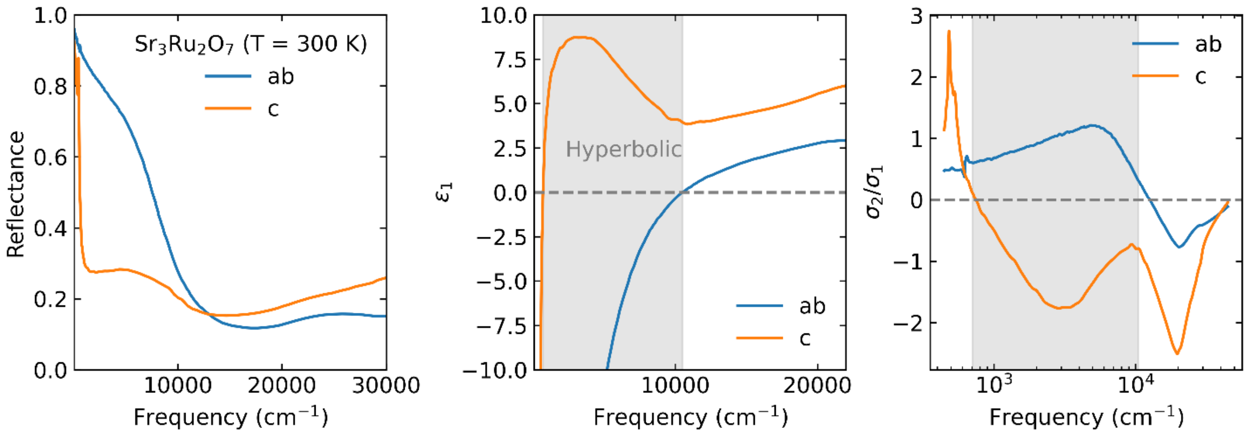

Fig. S24. $Sr_3Ru_2O_7$. Anisotropic reflectance, real part of the dielectric function and $\frac{\sigma_2}{\sigma_1}$ ratio of $Sr_3Ru_2O_7$ (*41*).

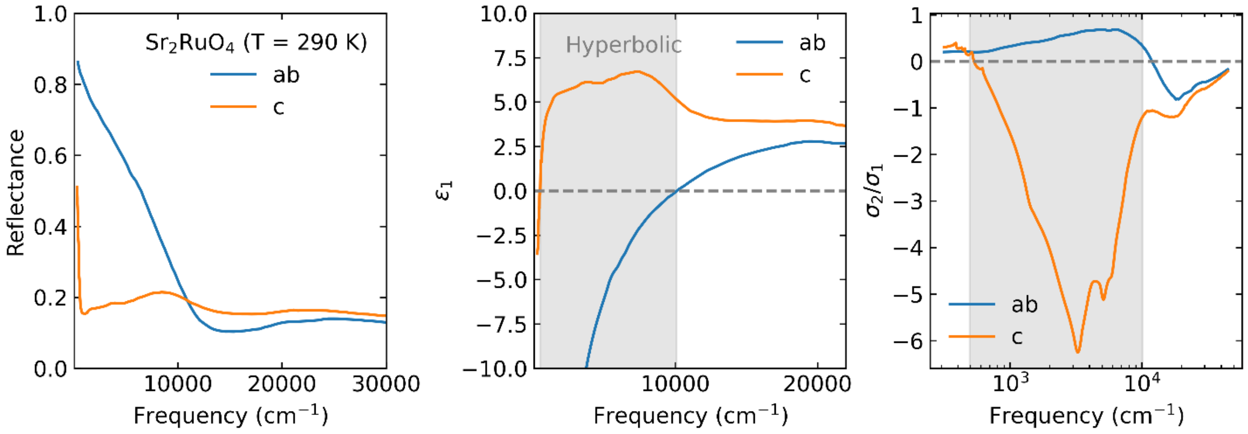

Fig. S25. Sr_2RuO_4. Anisotropic reflectance, real part of the dielectric function and $\frac{\sigma_2}{\sigma_1}$ ratio of Sr_2RuO_4 (*40*).

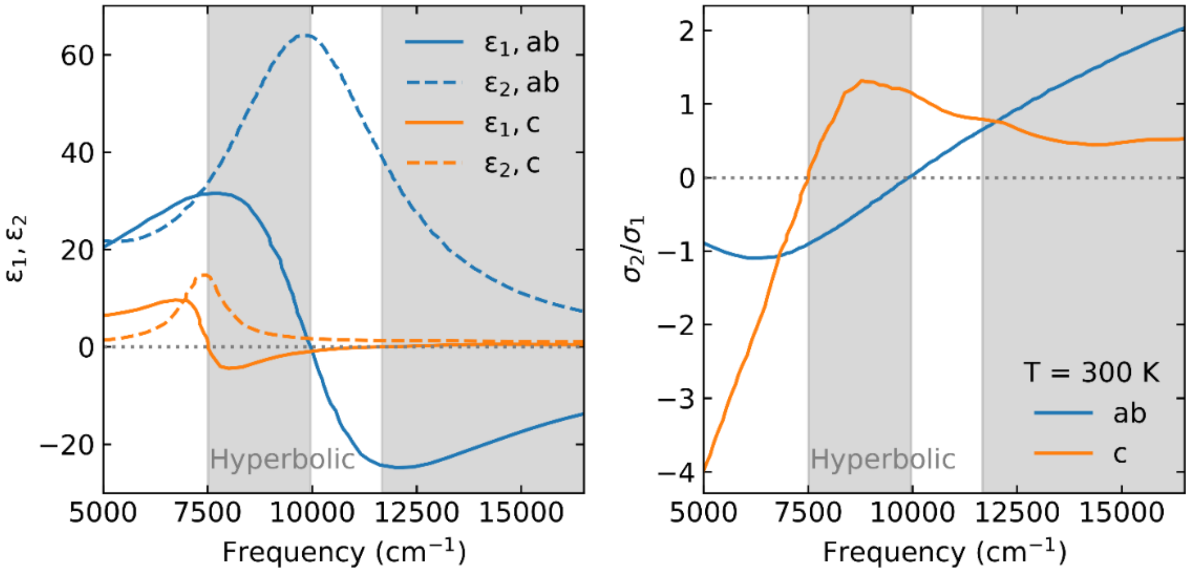

Fig. S26. Bi_2Te_3. Anisotropic dielectric function and $\frac{\sigma_2}{\sigma_1}$ ratio of Bi_2Te_3 (*42*).

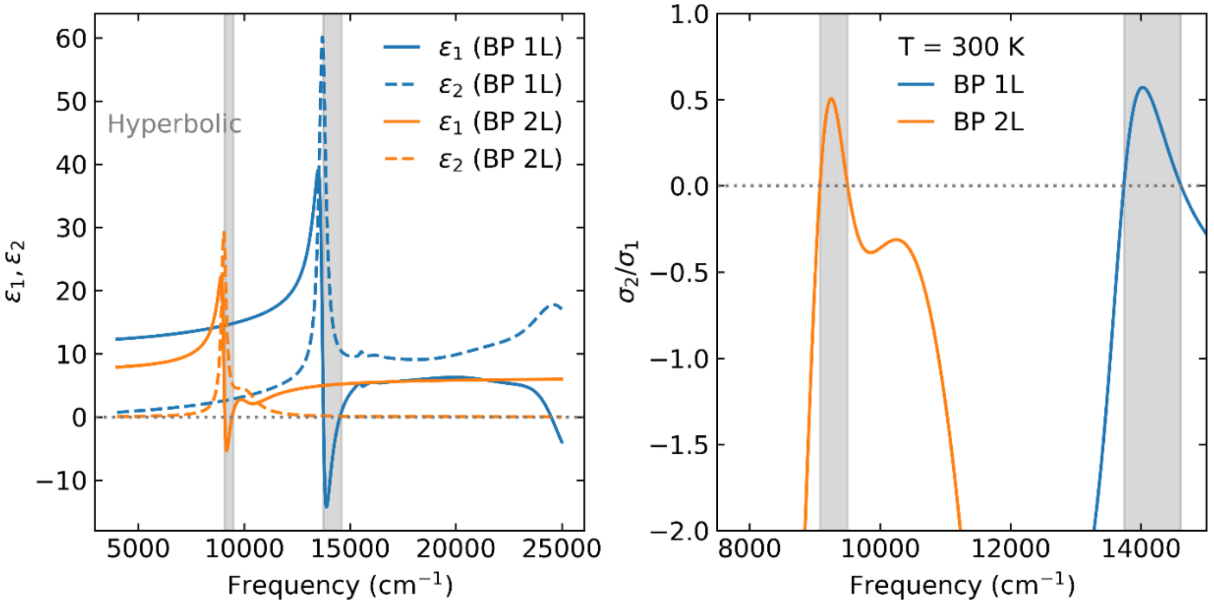

Fig. S27. Black phosphorus. Excitonic dielectric function of monolayer (1L) and bilayer (2L) black phosphorus along the arm-chair direction (*44*) and the corresponding $\frac{\sigma_2}{\sigma_1}$ ratio.

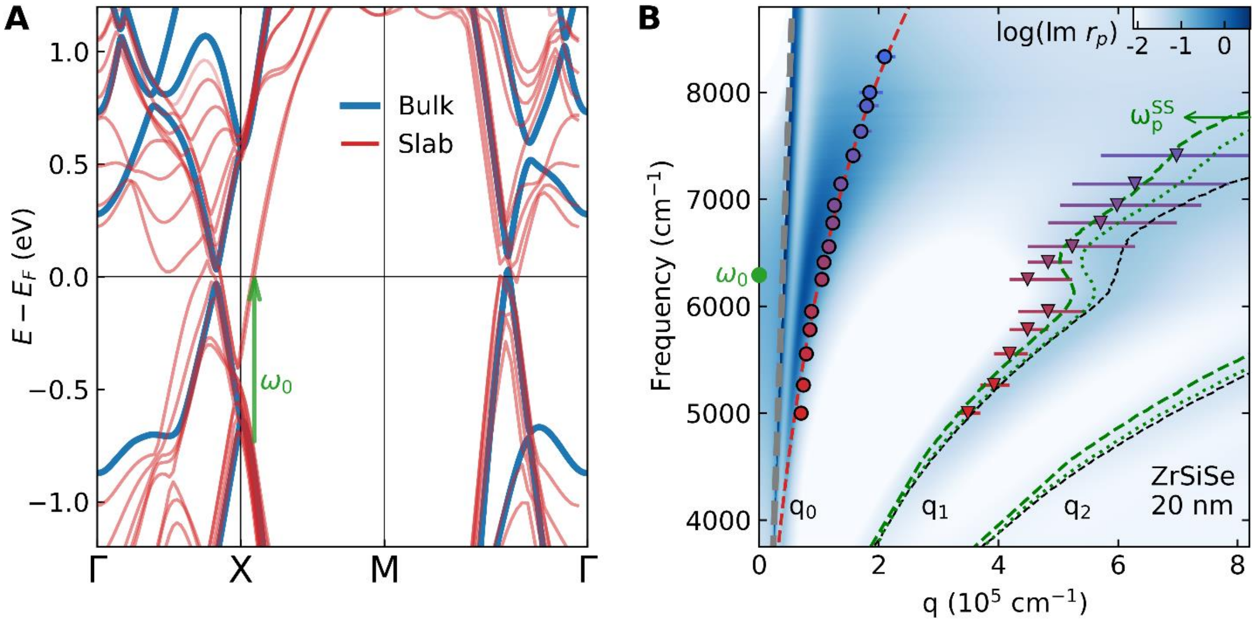

Fig. S28. Band structures of ZrSiSe and the impact of surface states on HPP dispersion. (**A**), First-principles calculations of the band structure for bulk ZrSiSe and a thin slab (5 layers) of ZrSiSe. Compared to the bulk bands, additional interband transitions associated with the surface states (e.g. green arrow) appear in the slab calculation. (**B**), Frequency-momentum dispersion of HPP plotted in the form of Im(r_p). Circles: the principal modes; triangles: higher-order polaritons. Data points are superimposed over the calculated Im(r_p) described in the main text. The grey dashed line represents the free-space light cone. Black dashed lines indicate numerical solutions for the divergence of Im(r_p) for the higher-order HPP branches using the dielectric functions in Fig. 2B. The red dashed line is a guide for the dispersion of the principal branch. Green dotted and dashed lines indicate the modified higher-order HPP dispersion with an interband resonance at $\omega_0 = 0.78$ eV and with increasing surface metallicity due to surface states ($\omega_p^{SS} = 1$ eV and $\omega_p^{SS} = 2$ eV for the green dotted and green dashed lines, respectively).

j	$\omega_{0,j}$ (cm^{-1})	$\omega_{p,j}$ (cm^{-1})	γ_j (cm^{-1})
1	0	5127.2	400.0
2	6291.3	2057.4	715.2
3	7367.8	2242.8	1055.3

Table S1. Parameters for the Drude-Lorentz model fitting of the experimental out-of-plane dielectric function of ZrSiSe using $\varepsilon_c(\omega) = \varepsilon_\infty + \sum_j \omega_{p,j}^2/(\omega_{0,j}^2 - \omega^2 - i\gamma_j\omega)$. Here, ε_∞=2.96 is the high frequency dielectric constant.

REFERENCES AND NOTES

1. L. M. Schoop, M. N. Ali, C. Straßer, A. Topp, A. Varykhalov, D. Marchenko, V. Duppel, S. S. P. Parkin, B. V. Lotsch, C. R. Ast, Dirac cone protected by non-symmorphic symmetry and three-dimensional Dirac line node in ZrSiS. *Nat. Commun.* **7**, 11696 (2016).

2. J. Hu, Z. Tang, J. Liu, X. Liu, Y. Zhu, D. Graf, K. Myhro, S. Tran, C. N. Lau, J. Wei, Z. Mao, Evidence of topological nodal-line fermions in ZrSiSe and ZrSiTe. *Phys. Rev. Lett.* **117**, 016602 (2016).

3. N. P. Armitage, E. J. Mele, A. Vishwanath, Weyl and Dirac semimetals in three-dimensional solids. *Rev. Mod. Phys.* **90**, 015001 (2018).

4. J. Orenstein, J. E. Moore, T. Morimoto, D. H. Torchinsky, J. W. Harter, D. Hsieh, Topology and symmetry of quantum materials via nonlinear optical responses. *Annu. Rev. Condens. Matter Phys.* **12**, 247–272 (2021).

5. Y. Shao, A. N. Rudenko, J. Hu, Z. Sun, Y. Zhu, S. Moon, A. J. Millis, S. Yuan, A. I. Lichtenstein, D. Smirnov, Z. Q. Mao, M. I. Katsnelson, D. N. Basov, Electronic correlations in nodal-line semimetals. *Nat. Phys.* **16**, 636–641 (2020).

6. M. B. Schilling, L. M. Schoop, B. V. Lotsch, M. Dressel, A. V. Pronin, Flat optical conductivity in ZrSiS due to two-dimensional Dirac bands. *Phys. Rev. Lett.* **119**, 187401 (2017).

7. T. Habe, M. Koshino, Dynamical conductivity in the topological nodal-line semimetal ZrSiS. *Phys. Rev. B.* **98**, 125201 (2018).

8. E. E. Narimanov, A. V. Kildishev, Naturally hyperbolic. *Nat. Photon.* **9**, 214–216 (2015).

9. H. N. S. Krishnamoorthy, Z. Jacob, E. Narimanov, I. Kretzschmar, V. M. Menon, Topological transitions in metamaterials. *Science* **336**, 205–209 (2012).

10. A. V. Kildishev, A. Boltasseva, V. M. Shalaev, Planar photonics with metasurfaces. *Science* **339**, 1232009 (2013).

11. G. Álvarez-Pérez, J. Duan, J. Taboada-Gutiérrez, Q. Ou, E. Nikulina, S. Liu, J. H. Edgar, Q. Bao, V. Giannini, R. Hillenbrand, J. Martín-Sánchez, A. Y. Nikitin, P. Alonso-González, Negative reflection of nanoscale-confined polaritons in a low-loss natural medium. *Sci. Adv.* **8**, eabp8486 (2022).

12. S. Dai, Q. Ma, T. Andersen, A. S. Mcleod, Z. Fei, M. K. Liu, M. Wagner, K. Watanabe, T. Taniguchi, M. Thiemens, F. Keilmann, P. Jarillo-Herrero, M. M. Fogler, D. N. Basov, Subdiffractional focusing and guiding of polaritonic rays in a natural hyperbolic material. *Nat. Commun.* **6**, 6963 (2015).

13. P. Li, M. Lewin, A. V. Kretinin, J. D. Caldwell, K. S. Novoselov, T. Taniguchi, K. Watanabe, F. Gaussmann, T. Taubner, Hyperbolic phonon-polaritons in boron nitride for near-field optical imaging and focusing. *Nat. Commun.* **6**, 7507 (2015).

14. S. Dai, Z. Fei, Q. Ma, A. S. Rodin, M. Wagner, A. S. McLeod, M. K. Liu, W. Gannett, W. Regan, K. Watanabe, T. Taniguchi, M. Thiemens, G. Dominguez, A. H. C. Neto, A. Zettl, F. Keilmann, P. Jarillo-Herrero, M. M. Fogler, D. N. Basov, Tunable phonon polaritons in atomically thin van der Waals crystals of boron nitride. *Science* **343**, 1125–1129 (2014).

15. A. J. Giles, S. Dai, I. Vurgaftman, T. Hoffman, S. Liu, L. Lindsay, C. T. Ellis, N. Assefa, I. Chatzakis, T. L. Reinecke, J. G. Tischler, M. M. Fogler, J. H. Edgar, D. N. Basov, J. D. Caldwell, Ultralow-loss polaritons in isotopically pure boron nitride. *Nat. Mater.* **17**, 134–139 (2018).

16. W. Ma, P. Alonso-González, S. Li, A. Y. Nikitin, J. Yuan, J. Martín-Sánchez, J. Taboada-Gutiérrez, I. Amenabar, P. Li, S. Vélez, C. Tollan, Z. Dai, Y. Zhang, S. Sriram, K. Kalantar-Zadeh, S.-T. Lee, R. Hillenbrand, Q. Bao, In-plane anisotropic and ultra-low-loss polaritons in a natural van der Waals crystal. *Nature* **562**, 557–562 (2018).

17. Z. Zheng, N. Xu, S. L. Oscurato, M. Tamagnone, F. Sun, Y. Jiang, Y. Ke, J. Chen, W. Huang, W. L. Wilson, A. Ambrosio, S. Deng, H. Chen, A mid-infrared biaxial hyperbolic van der Waals crystal. *Sci. Adv.* **5**, eaav8690 (2019).

18. J. Taboada-Gutiérrez, G. Álvarez-Pérez, J. Duan, W. Ma, K. Crowley, I. Prieto, A. Bylinkin, M. Autore, H. Volkova, K. Kimura, T. Kimura, M.-H. Berger, S. Li, Q. Bao, X. P. A. Gao, I. Errea, A. Y. Nikitin, R. Hillenbrand, J. Martín-Sánchez, P. Alonso-González, Broad spectral tuning of ultra-low-loss polaritons in a van der Waals crystal by intercalation. *Nat. Mater.* **19**, 964–968 (2020).

19. W. Ma, G. Hu, D. Hu, R. Chen, T. Sun, X. Zhang, Q. Dai, Y. Zeng, A. Alù, C.-W. Qiu, P. Li, Ghost hyperbolic surface polaritons in bulk anisotropic crystals. *Nature* **596**, 362–366 (2021).

20. N. C. Passler, X. Ni, G. Hu, J. R. Matson, G. Carini, M. Wolf, M. Schubert, A. Alù, J. D. Caldwell, T. G. Folland, A. Paarmann, Hyperbolic shear polaritons in low-symmetry crystals. *Nature* **602**, 595–600 (2022).

21. A. J. Sternbach, S. H. Chae, S. Latini, A. A. Rikhter, Y. Shao, B. Li, D. Rhodes, B. Kim, P. J. Schuck, X. Xu, X.-Y. Zhu, R. D. Averitt, J. Hone, M. M. Fogler, A. Rubio, D. N. Basov, Programmable hyperbolic polaritons in van der Waals semiconductors. *Science* **371**, 617–620 (2021).

22. A. J. Frenzel, C. C. Homes, Q. D. Gibson, Y. M. Shao, K. W. Post, A. Charnukha, R. J. Cava, D. N. Basov, Anisotropic electrodynamics of type-II Weyl semimetal candidate WTe2. *Phys. Rev. B.* **95**, 245140 (2017).

23. C. Wang, S. Huang, Q. Xing, Y. Xie, C. Song, F. Wang, H. Yan, Van der Waals thin films of WTe_2 for natural hyperbolic plasmonic surfaces. *Nat. Comm.* **11**, 1158 (2020).

24. Y. Shao, R. Jing, S. H. Chae, C. Wang, Z. Sun, E. Emmanouilidou, S. Xu, D. Halbertal, B. Li, A. Rajendran, F. L. Ruta, L. Xiong, Y. Dong, A. S. McLeod, S. S. Sunku, J. C. Hone, J. Moore, J. Orenstein, J. G. Analytis, A. J. Millis, N. Ni, D. Xiao, D. N. Basov, Nonlinear nanoelectrodynamics of a Weyl metal. *Proc. Natl. Acad. Sci. U.S.A.* **118**, e2116366118 (2021).

25. J. Ebad-Allah, S. Rojewski, M. Vöst, G. Eickerling, W. Scherer, E. Uykur, R. Sankar, L. Varrassi, C. Franchini, K.-H. Ahn, J. Kuneš, C. A. Kuntscher, Pressure-induced excitations in the out-of-plane optical response of the nodal-line semimetal ZrSiS. *Phys. Rev. Lett.* **127**, 076402 (2021).

26. S. Biswas, W. S. Whitney, M. Y. Grajower, K. Watanabe, T. Taniguchi, H. A. Bechtel, G. R. Rossman, H. A. Atwater, Tunable intraband optical conductivity and polarization-dependent epsilon-near-zero behavior in black phosphorus. *Sci. Adv.* **7**, eabd4623 (2021).

27. T. Low, A. Chaves, J. D. Caldwell, A. Kumar, N. X. Fang, P. Avouris, T. F. Heinz, F. Guinea, L. Martin-Moreno, F. Koppens, Polaritons in layered two-dimensional materials. *Nat. Mater.* **16**, 182–194 (2017).

28. A. Boltasseva, H. A. Atwater, Low-loss plasmonic metamaterials. *Science* **331**, 290–291 (2011).

29. J. D. Caldwell, A. V. Kretinin, Y. Chen, V. Giannini, M. M. Fogler, Y. Francescato, C. T. Ellis, J. G. Tischler, C. R. Woods, A. J. Giles, M. Hong, K. Watanabe, T. Taniguchi, S. A. Maier, K. S. Novoselov, Sub-diffractional volume-confined polaritons in the natural hyperbolic material hexagonal boron nitride. *Nat. Commun.* **5**, 5221 (2014).

30. S. Dai, Q. Ma, Y. Yang, J. Rosenfeld, M. D. Goldflam, A. McLeod, Z. Sun, T. I. Andersen, Z. Fei, M. Liu, Y. Shao, K. Watanabe, T. Taniguchi, M. Thiemens, F. Keilmann, P. Jarillo-Herrero, M. M. Fogler, D. N. Basov, Efficiency of launching highly confined polaritons by infrared light incident on a hyperbolic material. *Nano Lett.* **17**, 5285–5290 (2017).

31. J. A. Gerber, S. Berweger, B. T. O'Callahan, M. B. Raschke, Phase-resolved surface plasmon interferometry of graphene. *Phys. Rev. Lett.* **113**, 055502 (2014).

32. Z. Shi, H. A. Bechtel, S. Berweger, Y. Sun, B. Zeng, C. Jin, H. Chang, M. C. Martin, M. B. Raschke, F. Wang, Amplitude- and phase-resolved nanospectral imaging of phonon polaritons in hexagonal boron nitride. *ACS Photonics* **2**, 790–796 (2015).

33. S. Dai, W. Fang, N. Rivera, Y. Stehle, B.-Y. Jiang, J. Shen, R. Y. Tay, C. J. Ciccarino, Q. Ma, D. Rodan-Legrain, P. Jarillo-Herrero, E. H. T. Teo, M. M. Fogler, P. Narang, J. Kong, D. N. Basov, Phonon polaritons in monolayers of hexagonal boron nitride. *Adv. Mater.* **31**, 1806603 (2019).

34. Z. Fei, A. S. Rodin, G. O. Andreev, W. Bao, A. S. McLeod, M. Wagner, L. M. Zhang, Z. Zhao, M. Thiemens, G. Dominguez, M. M. Fogler, A. H. C. Neto, C. N. Lau, F. Keilmann, D. N. Basov, Gate-tuning of graphene plasmons revealed by infrared nano-imaging. *Nature* **487**, 82–85 (2012).

35. Z. Zhu, T.-R. Chang, C.-Y. Huang, H. Pan, X.-A. Nie, X.-Z. Wang, Z.-T. Jin, S.-Y. Xu, S.-M. Huang, D.-D. Guan, S. Wang, Y.-Y. Li, C. Liu, D. Qian, W. Ku, F. Song, H. Lin, H. Zheng, J.-F. Jia, Quasiparticle interference and nonsymmorphic effect on a floating band surface state of ZrSiSe. *Nat. Commun.* **9**, 4153 (2018).

36. S. Xue, M. Wang, Y. Li, S. Zhang, X. Jia, J. Zhou, Y. Shi, X. Zhu, Y. Yao, J. Guo, Observation of nodal-line plasmons in ZrSiS. *Phys. Rev. Lett.* **127**, 186802 (2021).

37. A. Topp, R. Queiroz, A. Grüneis, L. Müchler, A. W. Rost, A. Varykhalov, D. Marchenko, M. Krivenkov, F. Rodolakis, J. L. McChesney, B. V. Lotsch, L. M. Schoop, C. R. Ast, Surface floating 2D bands in layered nonsymmorphic semimetals: ZrSiS and related compounds. *Phys. Rev. X.* **7**, 041073 (2017).

38. J. Chen, M. Badioli, P. Alonso-González, S. Thongrattanasiri, F. Huth, J. Osmond, M. Spasenović, A. Centeno, A. Pesquera, P. Godignon, A. Zurutuza Elorza, N. Camara, F. J. G. de Abajo, R. Hillenbrand, F. H. L. Koppens, Optical nano-imaging of gate-tunable graphene plasmons. *Nature* **487**, 77–81 (2012).

39. M. N. Gjerding, M. Pandey, K. S. Thygesen, Band structure engineered layered metals for low-loss plasmonics. *Nat. Commun.* **8**, 15133 (2017).

40. C. Lewandowski, L. Levitov, Intrinsically undamped plasmon modes in narrow electron bands. *Proc. Natl. Acad. Sci. U.S.A.* **116**, 20869–20874 (2019).

41. N. F. Q. Yuan, H. Isobe, L. Fu, Magic of high-order van Hove singularity. *Nat. Commun.* **10**, 5769 (2019).

42. S. Tajima, G. D. Gu, S. Miyamoto, A. Odagawa, N. Koshizuka, Optical evidence for strong anisotropy in the normal and superconducting states in $Bi_2Sr_2CaCu_2O_{8+z}$. *Phys. Rev. B.* **48**, 16164–16167 (1993).

43. M. E. Berkowitz, B. S. Y. Kim, G. Ni, A. S. McLeod, C. F. B. Lo, Z. Sun, G. Gu, K. Watanabe, T. Taniguchi, A. J. Millis, J. C. Hone, M. M. Fogler, R. D. Averitt, D. N. Basov, Hyperbolic cooper-pair polaritons in planar graphene/cuprate plasmonic cavities. *Nano Lett.* **21**, 308–316 (2021).

44. T. Katsufuji, M. Kasai, Y. Tokura, In-plane and out-of-plane optical spectra of Sr_2RuO_4. *Phys. Rev. Lett.* **76**, 126–129 (1996).

45. C. Mirri, L. Baldassarre, S. Lupi, M. Ortolani, R. Fittipaldi, A. Vecchione, P. Calvani, Anisotropic optical conductivity of $Sr_3Ru_2O_7$. *Phys. Rev. B.* **78**, 155132 (2008).

46. M. Esslinger, R. Vogelgesang, N. Talebi, W. Khunsin, P. Gehring, S. de Zuani, B. Gompf, K. Kern, Tetradymites as natural hyperbolic materials for the near-infrared to visible. *ACS Photonics* **1**, 1285–1289 (2014).

47. G. V. Naik, J. Liu, A. V. Kildishev, V. M. Shalaev, A. Boltasseva, Demonstration of Al:ZnO as a plasmonic component for near-infrared metamaterials. *Proc. Natl. Acad. Sci. U.S.A.* **109**, 8834–8838 (2012).

48. F. Wang, C. Wang, A. Chaves, C. Song, G. Zhang, S. Huang, Y. Lei, Q. Xing, L. Mu, Y. Xie, H. Yan, Prediction of hyperbolic exciton-polaritons in monolayer black phosphorus. *Nat. Comm.* **12**, 5628 (2021).

49. B. Xu, Y. M. Dai, L. X. Zhao, K. Wang, R. Yang, W. Zhang, J. Y. Liu, H. Xiao, G. F. Chen, A. J. Taylor, D. A. Yarotski, R. P. Prasankumar, X. G. Qiu, Optical spectroscopy of the Weyl semimetal TaAs. *Phys. Rev. B.* **93**, 121110 (2016).

50. C. J. Tabert, J. P. Carbotte, Optical conductivity of Weyl semimetals and signatures of the gapped semimetal phase transition. *Phys. Rev. B.* **93**, 085442 (2016).

51. A. Sommerfeld, O. Laporte, P. A. Moldauer, Optics: Vol. 5 of lectures on theoretical physics. *Phys. Today* **8**, 16 (1955).

52. P. C. Clemmow, A method for the exact solution of a class of two-dimensional diffraction problems. *Proc. R. Soc. Lond. A.* **205**, 286–308 (1951).

53. N. Tancogne-Dejean, A. Rubio, Parameter-free hybridlike functional based on an extended Hubbard model: DFT+U+V. *Phys. Rev. B.* **102**, 155117 (2020).

54. N. Tancogne-Dejean, M. J. T. Oliveira, X. Andrade, H. Appel, C. H. Borca, G. le Breton, F. Buchholz, A. Castro, S. Corni, A. A. Correa, U. de Giovannini, A. Delgado, F. G. Eich, J. Flick, G. Gil, A. Gomez, N. Helbig, H. Hübener, R. Jestädt, J. Jornet-Somoza, A. H. Larsen, I. V. Lebedeva, M. Lüders, M. A. L. Marques, S. T. Ohlmann, S. Pipolo, M. Rampp, C. A. Rozzi, D. A. Strubbe, S. A. Sato, C. Schäfer, I. Theophilou, A. Welden, A. Rubio, Octopus, a computational framework for exploring light-driven phenomena and quantum dynamics in extended and finite systems. *J. Chem. Phys.* **152**, 124119 (2020).

55. G. Gatti, A. Crepaldi, M. Puppin, N. Tancogne-Dejean, L. Xian, U. De Giovannini, S. Roth, S. Polishchuk, Ph. Bugnon, A. Magrez, H. Berger, F. Frassetto, L. Poletto, L. Moreschini, S. Moser, A. Bostwick, E. Rotenberg, A. Rubio, M. Chergui, M. Grioni, Light-induced renormalization of the Dirac quasiparticles in the nodal-line semimetal ZrSiSe. *Phys. Rev. Lett.* **125**, 076401 (2020).

56. C. Hartwigsen, S. Goedecker, J. Hutter, Relativistic separable dual-space Gaussian pseudopotentials from H to Rn. *Phys. Rev. B.* **58**, 3641–3662 (1998).
